# Neural Melanocortin Receptors in Fish: Insights into Growth Regulation and Aquaculture Advancement

**DOI:** 10.3390/biom16060839

**Published:** 2026-06-08

**Authors:** Ren-Lei Ji, Ya-Xiong Tao

**Affiliations:** Department of Anatomy, Physiology and Pharmacology, College of Veterinary Medicine, Auburn University, Auburn, AL 36849, USA; rlj0027@auburn.edu

**Keywords:** melanocortin receptor, MC3R, MC4R, aquaculture industry, growth performance, energy homeostasis, hypothalamus

## Abstract

Understanding and regulating fish growth is vital for the economic sustainability of aquaculture. The melanocortin-3 and -4 receptors (MC3R/MC4R, known as neural MCRs), integral components of the leptin–melanocortin circuit, play crucial roles in vertebrate energy homeostasis and growth. Abnormal neural MCR signaling contributes to human obesity. In teleosts, Mc4r was first comprehensively studied in goldfish in 2003. Since then, Mc4r has been characterized in various teleosts. Genetic and pharmacological reduction of neural Mcr signaling can increase feeding or growth in several fish models, although its aquaculture value must be evaluated using production endpoints such as feed conversion, body composition, reproduction, welfare, and biosafety. Furthermore, neural Mcrs also play a role in modulating reproductive processes and sexual function in teleosts. This review systematically examines recent progress on the roles of fish neural Mcrs, offering an overview of basic molecular characteristics, tissue distribution, and pharmacology. Physiological roles and mechanisms in growth regulation are reviewed. Finally, the potential and limitations of targeting neural Mcrs for aquaculture-relevant traits are discussed. This work contributes to our understanding of the evolution of energy homeostasis regulation in vertebrates, providing a foundation for healthier and more efficient aquaculture practices.

## 1. Introduction

A comprehensive understanding of energy homeostasis in cultured aquatic animals is crucial for the sustainable advancement of aquaculture. This field seeks to optimize the conversion of feed into healthy fish, producing high-quality, nutritious products for human consumption. Efforts to enhance growth and feed efficiency currently rely heavily on the time-consuming method of traditional selective breeding. Therefore, investigating the hormonal control of energy metabolism in commercially significant cultured aquatic animals holds great potential. Such insights can lead to the development of innovative approaches that markedly improve growth and feed efficiency, resulting in superior economic returns and promoting the sustainability of aquaculture practices.

The melanocortin system comprises five receptors, designated MC1R to MC5R based on the sequence of their identification, and six endogenous ligands (two antagonists and four agonists). The agonists, α-, β-, and γ-melanocyte-stimulating hormone (MSH) and adrenocorticotropic hormone (ACTH) originate from the tissue-specific post-translational cleavage of proopiomelanocortin (POMC) [1,2]. The melanocortin system stands out for having two unique endogenous antagonists: agouti-related peptide (AgRP) and agouti or agouti-signaling protein (ASIP) [3]. Among the 800 G protein-coupled receptors (GPCRs) found in humans, these endogenous antagonists were the only known for many years until another antagonist was identified for ghrelin receptor, LEAP2 (liver-expressed antimicrobial peptide 2) [4].

MC1R, known as the MSH receptor for decades, is highly expressed in the hair follicles and skin, regulating pigmentation [5,6]. MC2R (previously known as the ACTH receptor) is highly expressed in the adrenal cortex and plays a crucial role in controlling adrenal steroid production [7]. MC5R is broadly distributed, with notable expression in exocrine glands, regulating exocrine gland secretions [8]. MC3R and MC4R, referred to as neural MCRs, are predominantly found in the central nervous system (CNS) [9,10,11,12] and are crucial for maintaining energy balance [13,14,15,16,17].

**Figure 1:**
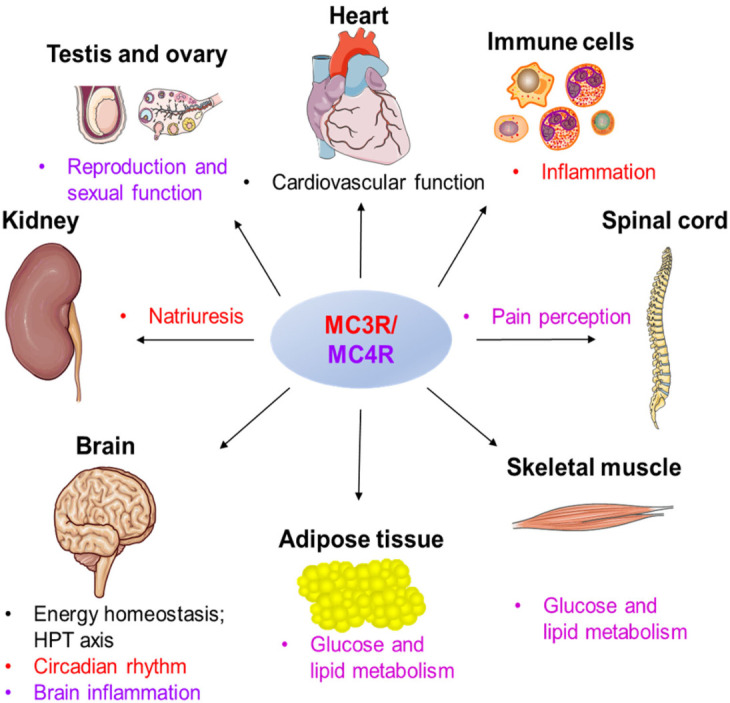
Pleiotropic functions of mammalian MC3R and MC4R and their relevance as a comparative framework for fish neural Mcr studies. The red font represents MC3R function, and the purple font represents MC4R function. The black font represents functions for both MC3R and MC4R. This figure summarizes functions identified largely from mammalian studies and is intended as a comparative framework for fish neural Mcr research. In fish, current evidence most strongly supports roles of Mc3r/Mc4r in feeding, growth, lipid/glucose metabolism, and reproduction, whereas functions such as cardiovascular regulation, renal function, inflammation, and skeletal muscle regulation remain less well established or require further investigation. HPT: hypothalamus–pituitary–thyroid. Figure reprinted from Ref. [17] with permission from Elsevier.

Neural MCRs possess unique and non-overlapping ways of regulating energy balance [18,19,20,21,22]. Specifically, MC3R primarily governs feeding rhythm and feed efficiency, whereas MC4R is crucial for regulating both dietary intake and energy consumption. Deleting *Mc3r* and/or *Mc4r* in mice leads to the development of obesity. Mutations in these receptors in humans are associated with monogenic obesity [15,16,17,23,24,25,26,27]. Furthermore, based largely on mammalian studies, MC3R and MC4R have been implicated in several additional physiological processes, including inflammation, cardiovascular regulation, as well as reproductive and sexual function (Figure 1) [15,17,28,29,30,31,32,33,34,35,36].

Neural Mcrs have also been studied in fish (Table 1 and Table 2), though the extent to which mammalian MC3R/MC4R functions are conserved in fish remains incompletely resolved and should be evaluated in a species-specific manner. Fish *mc3r* and *mc4r* were first cloned from zebrafish (*Danio rerio*) in the 2000s [37,38]. Since then, these two genes have been identified and characterized in both teleosts and cartilaginous fish (Table 1 and Table 2). These receptors are important regulators of energy homeostasis. Thus, activating these receptors with agonists decreases food consumption in both goldfish (*Carassius auratus*) and rainbow trout (*Oncorhynchus mykiss*), while antagonist administration results in increased food intake [39,40,41,42]. Teleost Mc3r/Mc4r are also essential for the regulation of reproduction. Understanding how neural MCRs regulate energy homeostasis is essential for aquatic animals. These insights may help guide future strategies to improve growth, feed efficiency, and product quality in cultured aquatic species, provided that species-specific efficacy and safety are carefully evaluated.

This review aims to comprehensively summarize the latest research findings on the functions and regulatory mechanisms of Mc3r and Mc4r in growth regulation in fish. Special attention is given to their roles in controlling feeding and energy metabolism. Furthermore, potential applications of Mc3r and Mc4r in the aquaculture industry are discussed, with the goal of providing insights for further research and promoting their practical implementation in aquaculture.

## 2. Fish Neural MCR Genes and Tissue Expression

### 2.1. Mammalian Neural MCR Genes

Human (h) *MC3R* and *MC4R* were initially cloned in 1993. *MC4R* encodes a 332-amino-acid protein from an intronless gene at chromosome 18q21.3 [11,12], and *MC3R* is located on 20q13.2, consisting of a single exon and encoding a protein of 360 amino acids [9]. The two receptors share approximately 61% sequence similarity, but mediate distinct physiological functions.

**Figure 2:**
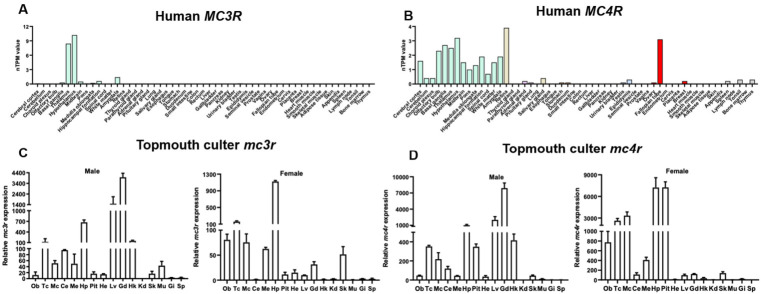
Gene expression of *MC3R* and *MC4R* in various tissues in both humans and fish. Human data were based on the Human Protein Atlas (https://www.proteinatlas.org/; accessed on 25 May 2024, Ref. [87]). nTPM indicates normalized protein-coding transcripts per million. Color coding is based on tissue groups with functional features in common. Fish data reproduced from Refs. [62,76] The mRNA levels of *mc3r* and *mc4r* were measured by qRT-PCR. Data are presented as the mean ± SEM. Mc: mesencephalon; Ob: olfactory bulb; Ce: cerebellum; Tc: telencephalon; Hp: hypothalamus; Me: medulla; Pit: pituitary gland; Lv: liver; He: heart; St: stomach; Kd: kidney; Int: intestine; Hk: head kidney; Gd: gonad; Mu: muscle; Sk: skin; Gi: gill; Sp: spleen.

In mammals, MC3R and MC4R are expressed predominantly in the central nervous system, particularly in hypothalamic and other neural circuits involved in feeding, energy expenditure, metabolic regulation, and neuroendocrine control. Although both receptors have also been detected in peripheral tissues [9,32,33,82,83] (Figure 2), their best-established functions are centered on energy homeostasis [10,11,12,84].

**Figure 3:**
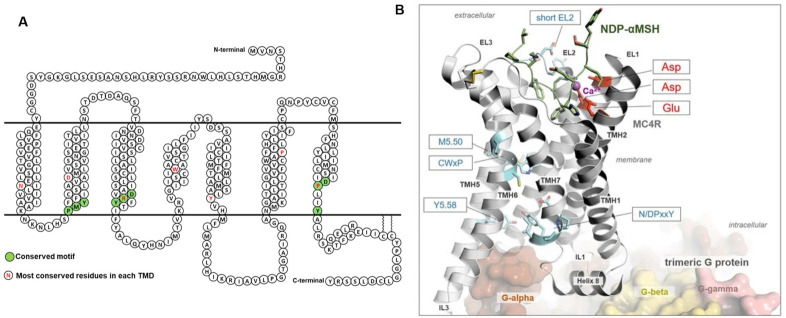
Schematic model and structure of MC4R. (**A**) Schematic model; (**B**) structure. The determined structure of hMC4R in complex with binding partners in an active state (PDB ID: 7PIV) [88] reveals detailed insights into binding by ligand, G protein, and calcium. Calcium serves as an essential co-factor for peptide ligand binding, as demonstrated by multiple ligand–MCR complex structures. Additionally, amino-acid side chains involved in signaling regulation and transduction of MCRs, including the CWxP and N/DxxY motifs common to most class A GPCRs, are highlighted in stick representation. Notably, a short EL2 is present in all vertebrate MCRs, which likely impairs the competitive binding and alignment of POMC-derived ligands. This structural model was generated using the PyMol molecular graphics system, version 2.5.5 (Schrödinger, LLC, New York, NY, USA). Reprinted with permission from Ref. [89]. Copyright 2024.

**Figure 4:**
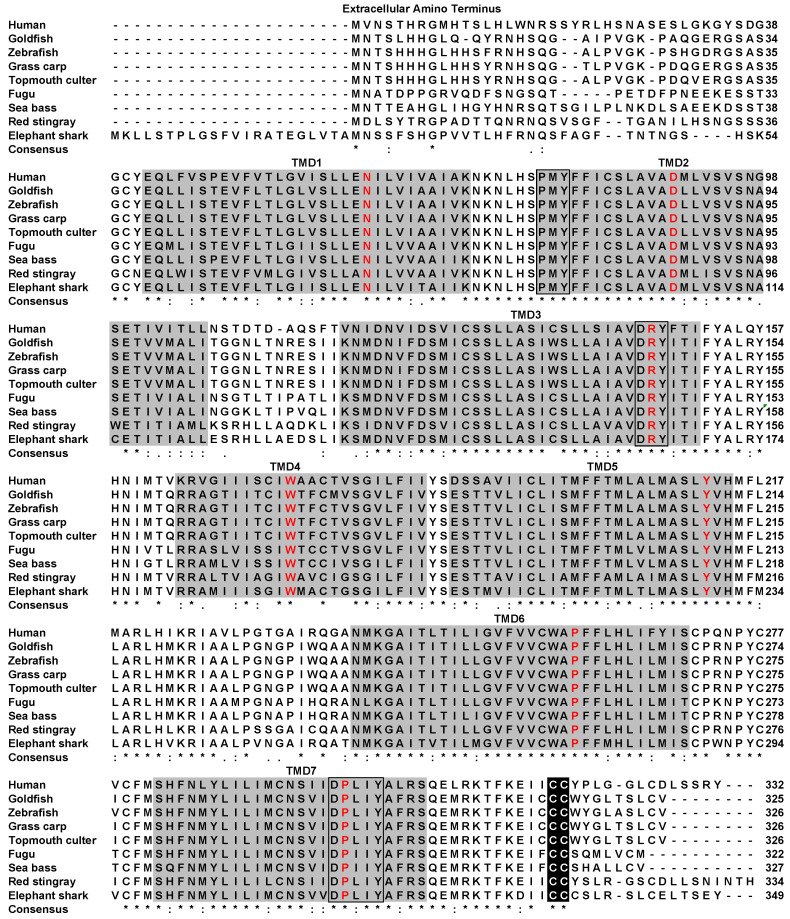
Comparison of amino-acid sequences of MC4Rs among different species. Transmembrane domains (TMDs) are shown in shaded boxes, with the most conserved residues in each TMD shown in red. PMY, DRY, DPxxY motifs are indicated by open boxes. The potential palmitoylation sites at the C termini are indicated by black boxes. Asterisks (*) indicate the same amino acids.

**Figure 5:**
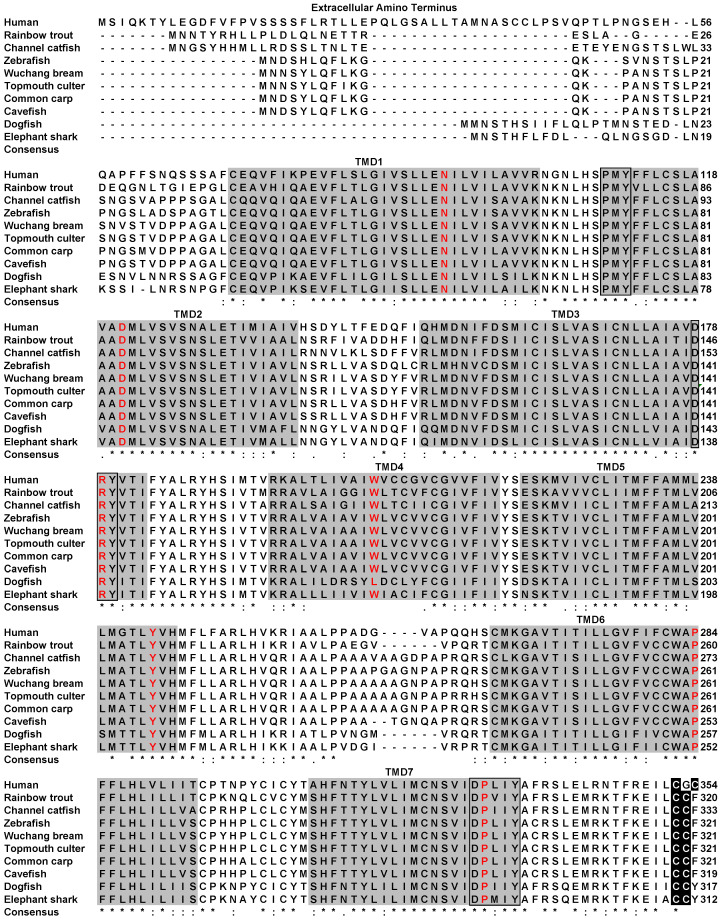
Comparison of amino-acid sequences of MC3Rs among different species. Transmembrane domains (TMDs) are shown in shaded boxes, with the most conserved residues in each TMD shown in red. PMY, DRY, and DPxxY motifs are shown in open boxes. The potential palmitoylation sites at the C termini are indicated by black boxes. Asterisks (*) indicate the same amino acids.

Structurally, MC3R and MC4R contain conserved class A GPCR motifs, including the DRY motif in transmembrane domain 3 and the DPxxY motif in transmembrane domain 7, as well as the melanocortin receptor-specific PMY motif in transmembrane domain 2. Compared with many other class A GPCRs, MCRs have relatively short extracellular and intracellular loops, particularly an extremely short extracellular loop 2 (Figure 3, Figure 4 and Figure 5). This mammalian framework provides a useful reference point for the fish-centered discussion below, where fish Mc3r and Mc4r show broader tissue distribution, divergent pharmacology, and distinct potential relevance to aquaculture.

### 2.2. Fish Neural MCR Genes

In fish, the *mc4r* gene was first cloned from zebrafish in 2002 [37], and was initially comprehensively studied in goldfish [40]. Subsequently, Mc4r has been characterized in both teleosts and cartilaginous fish (Table 1). Fish *mc4r* has been identified in all fish studied, and is found in 211 fish species listed in NCBI (https://www.ncbi.nlm.nih.gov/gene/?term=mc4r+fish, accessed on 25 May 2024).

The fish *mc3r* gene was initially identified in zebrafish in 2003 [38]. Subsequently, *mc3r* has been identified in several teleosts (Table 2), such as zebrafish [43,81,85], channel catfish (*Ictalurus punctatus*) [75], Wuchang bream (*Megalobrama amblycephala*) [74], topmouth culter (*Culter alburnus*) [76], cavefish (*Onychostoma macrolepis*) [77], rainbow trout [78], grass carp (*Ctenopharyngodon idella*) [80], common carp (*Cyprinus carpio*) [79], and red crucian carp (*Carassius auratus red var*.) [70]. Cartilaginous fish have also been studied (Table 2), including spiny dogfish (*Squalus acanthias*) [81], stingray (*Dasyatis akajei*) [72], and elephant shark (*Callorhynchus milii*) [73]. Of note, unlike fish *mc4r*, the *mc3r* gene is not widely distributed among teleosts, being found in only 61 fish species listed in NCBI (https://www.ncbi.nlm.nih.gov/gene/?term=mc3r+fish) (accessed on 25 May 2024) (Table S2) and not found in several species, including Yangtze sturgeon (*Acipenser dabryanus*), cichlid (*Simochromis diagramma*), ricefield eel (*Monopterus albus)*, American paddlefish (*Polyodon spathula*), fugu (*Takifugu rubripes*), medaka (*Oryzias latipes*), stickleback (*Gasterosteus aculeatus)*, and orange-spotted grouper (*Epinephelus coioides*) [38,56,58,76,81,86].

Compared with mammalian neural MCR genes, fish *mc3r* and *mc4r* genes show broader expression, spanning from the central nervous system to peripheral tissues, as evidenced by studies across various tissues, such as brain, intestine, kidney, liver, spleen, heart, and muscles (Table 1 and Table 2 and Figure 2). Importantly, both *mc3r* and *mc4r* expression have sexual dimorphism in fish, such as being expressed in the testis, but not in the ovary (Table 1 and Table 2 and Figure 2) [76]. The physiological roles of the neural Mcrs in these tissues are not fully studied.

Much like in mammals [90,91,92,93], fish *mc3r* and *mc4r* genes are expressed during embryonic development. In zebrafish, both *mc3r* and *mc4r* are expressed between 2 and 7 days post-fertilization [43]. Similarly, in topmouth culter, both *mc3r* and *mc4r* are detected from 1 to 5 days post-fertilization [76].

Fish Mc3r and Mc4r share substantial homology with MC3R and MC4R from other vertebrates, showing over 70% similarity to mammalian counterparts, with classical characteristics of MCRs, such as ICLs, ECLs, and seven putative TMDs (Figure 4 and Figure 5). The predicted amino-acid sequences within the TMDs of fish Mc3r/Mc4r are highly conserved across species (Figure 4 and Figure 5). Notably, the DRY, PMY, and DPxxY motifs of fish Mc3r/Mc4r align with homologous positions in the MC3Rs/MC4Rs of other species. Additionally, the C termini of fish Mc3r/Mc4r include the consensus sequence for phosphorylation by protein kinase C (Thr–Phe–Lys).

The available data indicate that fish neural Mcrs retain the core structural features of class A GPCRs, but their genes can differ in copy number and tissue distribution, with potential physiological function differences in the receptors. The broad peripheral expression of fish *mc3r* and *mc4r* should not be interpreted simply as a conserved extension of mammalian central melanocortin biology. Rather, it suggests that fish neural Mcrs may have been adapted to coordinate central appetite control with tissue-level regulation of metabolism, reproduction, stress responsiveness, and developmental physiology in a species- and context-dependent manner. Future studies should move beyond descriptive cloning and expression profiling toward receptor-function mapping in aquaculture-relevant species, with particular attention to gene duplication, developmental stage, sex, nutritional state, and tissue-specific signaling context.

## 3. Physiology of Neural MCRs

### 3.1. Energy Balance

The central melanocortin system consists of a network of CNS circuits that involve various types of neurons expressing either the ligands or the receptors. Two distinct populations of neurons in the ARC produce endogenous ligands for the neural MCRs. Specifically, one subset, situated in the lateral part of the ARC, expresses POMC. Another subset, found in the medial ARC, co-expresses AgRP and neuropeptide Y [94,95]. POMC neurons reduce food intake and enhance energy expenditure by releasing α-MSH, whereas AgRP neurons exert antagonistic roles. Both AgRP and POMC neurons are classified as “first-order” neurons, which can detect and integrate external stimulation, including nutrient and humoral cues, such as insulin, leptin, serotonin, orexin, ghrelin, and glucose [96,97,98,99,100,101,102,103]. Of these, leptin is one of the most thoroughly investigated hormones, with a key functional role in regulating energy balance and metabolism.

Neurons expressing MC3R and MC4R in numerous brain regions are targeted by α-MSH and AgRP, categorized as “second-order” neurons [104]. Leptin stimulates POMC neurons to promote release of α-MSH and suppresses AgRP neurons to decrease AgRP production. Activation of neural MCRs in these neurons by α-MSH induces negative energy balance, while inhibition by AgRP promotes positive energy balance [104]. This leptin-regulated melanocortin pathway plays a crucial role in both rodents and humans, with variants in its components contributing to obesity and various metabolic disorders [23,24,105,106,107,108,109,110,111].

**Table 3:** Representative functional evidence linking fish melanocortin-system components to feeding, growth, and aquaculture-relevant traits.

Species	Target/Intervention	Feed Intake	Growth/Body-Weight Phenotype	SGR/FCR/Protein Efficiency	Body Composition/Metabolism	Reproduction/Health/Welfare Endpoints	Experimental Duration/ Culture Condition	Reference
Goldfish	Central pharmacological modulation of Mc4r signaling: MTII or NDP-MSH; HS024	MTII or NDP-MSH suppressed food intake; HS024 increased food intake	NR	NR	NR	NR	Acute intracerebroventricular injection/feeding assay	[40,41]
Rainbow trout	Central pharmacological modulation of neural Mcr signaling: MTII, HS024, SHU9119	MTII reduced food intake; HS024 and SHU9119 increased food intake	NR	NR	NR	NR	Acute pharmacological feeding/motivation assay	[42]
Zebrafish	*agrp* overexpression or loss-of-function/knockout	NR	*agrp* overexpression increases linear growth; *agrp* loss reduced growth	NR	*agrp* overexpression increases adipocyte hypertrophy	NR	Transgenic/genetic model; developmental growth assessment	[112,113]
Zebrafish	*asip1* overexpression	NR	Delayed early growth but enhanced linear growth after puberty	NR	NR	Increased egg production, reduced spawning frequency, and lower hatching rate	Transgenic model; developmental and reproductive assessment	[114]
Zebrafish	*mrap2a* or *mrap2b* knockout	NR	*mrap2a* knockout decreases larval growth; *mrap2b* knockout inhibites adult growth	NR	NR	Developmental-stage-specific effects; broader welfare endpoints not reported	Genetic knockout model; larval and adult stages	[115]
*Xiphophorus*	Nonfunctional/dominant-negative *mc4r* B alleles	NR	Larger male body size associated with nonfunctional *mc4r* alleles	NR	NR	Delayed puberty onset; effects on mating behavior and reproductive tactics	Natural genetic variation/genotype–phenotype association; functional receptor characterization	[48,116]
Mexican cavefish	Natural loss-of-function mc4r allele (G145S)	Enhanced appetite	Increased growth/body size	NR	Resistance to starvation; obesity-like energy-storage phenotype	NR	Surface and cavefish comparative genetic model; nutrient-poor adaptation context	[52]
Channel catfish	CRISPR–Cas9-edited *mc4r* mutation	NR	Greater growth at multiple life stages; homozygous/biallelic mutants reached market size faster; increased body weight reported	Lower FCR reported in edited fish; SGR and protein efficiency not consistently reported	Not reported	Reproduction, health, and welfare endpoints not fully reported	Tank and pond culture conditions; life-stage and market-size assessments	[65,66]
Red crucian carp	CRISPR–Cas9-generated *mc4r*^+/−^ fish	Increased food intake	Increased fish length, body weight, and body depth	NR	Increased visceral fat mass; altered liver and muscle transcriptomic pathways related to glucose/lipid metabolism	Reproductive endpoints not comprehensively assessed	Heterozygous gene-edited model; controlled experimental rearing	[70]
Red crucian carp	CRISPR–Cas9-generated *mc3r*^+/−^ fish	NR	Slightly increased fish length and body depth; no significant effect on body weight	NR	Modest increase in visceral fat mass; altered liver and muscle transcriptomic pathways related to glucose/lipid metabolism	Reproductive endpoints not comprehensively assessed	Heterozygous gene-edited model; controlled experimental rearing	[70]
Spotted scat	Mc4r agonists/antagonists: THIQ, NDP-MSH, SHU9119, Ipsen 5i	NR	NR	NR	NR	Altered reproductive gene expression, including fshb, lhb, and gnrh-related responses	In vitro hypothalamic incubation and in vivo injection	[54]
Black rockfish	α-MSH and β-MSH treatment/Mc4r-related reproductive signaling	NR	NR	NR	NR	Altered brain reproductive neuropeptide expression and ovarian steroidogenic gene expression	Reproductive tissue/gene-expression study	[61]

NR, not reported.

MC4R is the central melanocortin system’s most thoroughly investigated target. *Mc4r^−/−^* mice have an obese phenotype with reduced energy expenditure and elevated food intake [18,21]. Even heterozygous mice have greater body weight than their wild-type (WT) littermates [18]. The *Mc4r*^−/−^ mice show no response to melanotan II (MTII, non-selective superpotent agonist for MC4R) regarding food intake or energy expenditure [117,118]. Further investigations have unveiled that MC4R in amygdala and paraventricular nucleus neurons regulate food intake, whereas other neurons expressing MC4R regulate energy expenditure [21]. MC4R in POMC neurons could function as both an auto-excitatory and auto-potentiation mechanism, promoting POMC neuron activation to regulate energy balance [119]. Furthermore, MC4R is necessary for leptin’s inhibitory effect on food intake [117]. Over 300 mutations have been discovered in human *MC4R*, establishing *MC4R* variants as the predominant cause of monogenic obesity [17,26,120,121,122].

Although the roles of MC3R in energy balance have not been studied as extensively as those of MC4R, MC3R still plays a critical role in regulating energy balance [19,20]. Unlike *Mc4r* deletion, which leads to hyperphagia in mice, the deletion of *Mc3r* results in a reduction in lean mass and an increase in fat mass, with normal food consumption or hypophagia, highlighting the importance of MC3R in regulating feed efficiency. *Mc3r*^−/−^ mice have markedly reduced fatty acid oxidation. This is likely a result of a delicate imbalance between fat oxidation and intake [19,20,123]. Mice lacking *Mc3r* show altered metabolic response [124], impaired behavioral adaptation [125], and dysregulated rhythmic expression of clock genes [126], indicating functional roles of MC3R in modulating circadian rhythm. It also regulates energy rheostasis [127]. MC3R in POMC neurons has been recognized and suggested to function as an auto-inhibitory factor, dampening the activation of POMC neurons to modulate energy balance [98,128]. Hence, MC3R plays a distinct function in modulating energy balance compared to MC4R. This includes functions such as adapting to fasting, regulating feed efficiency, and sustaining circadian rhythm.

In fish, the initial study confirming the involvement of Mcrs in energy homeostasis was conducted in goldfish in 2003 [40,45] (Table 3). Intracerebroventricular (ICV) injection of the non-selective MC4R agonists MTII or [Nle^4^, D-Phe^7^]-α-MSH (NDP-MSH) suppresses food intake, whereas HS024, an MC4R-specific antagonist, enhances food consumption [40,41]. The anorexigenic effect of octadecaneuropeptide is also influenced by Mc4r (action decreased by HS024) [129]. Similar findings were observed in rainbow trout, where MTII reduces food intake while HS024 and the MC3R/MC4R antagonist SHU9119 increases food intake [42]. Increased expression of *agrp1* (but not *agrp2*) was suggested to account for the increased food intake in growth hormone-transgenic common carp [130]. Fasting in Atlantic salmon increases hypothalamic *agrp1* (but not *agrp2*) and deceases *pomca2* expression, suggesting that Agrp1 is an orexigenic signal in Atlantic salmon [131].

Several studies have linked fish *mc4r* mutations or single-nucleotide polymorphisms (SNPs) to fish growth performance, further underlining the essential role of Mc4r in modulating energy balance in fish [48,52] (Table 3). The initial report originated from *Xiphophorus* fish, where two mutant alleles (B1 and B2) defective in signaling were linked to a larger body and the mutant alleles exert dominant negative effect on the wild-type (WT) allele [48,116]. In Mexican cavefish (*Astyanax mexicanus*), a loss-of-function mutant Mc4r (reduced constitutive activity and ligand-induced cyclic adenosine monophosphate (cAMP) signaling) contributes to enhanced appetite, growth, and resistance to starvation [52]. These findings indicate that impaired Mc4r signaling in fish may be the cause of their hyperphagia and obesity-like traits (see discussion below).

The role of AgRP is also preserved in lower vertebrates. Agrp acts as an inverse agonist for fish neural Mcrs. Transgenic zebrafish overexpressing *agrp*, leading to decreased Mcr signaling, have increased adipocyte hypertrophy and linear growth [112]. Conversely, *agrp* knockout in zebrafish results in decreased fish growth [113]. ASIP is another endogenous antagonist for MCRs. Zebrafish overexpressing *asip1*, leading to decreased activity of Mc4r, show no alteration in the timing of puberty, but notably show delays in early growth. However, these fish exhibit enhanced linear growth after completing puberty [114]. Mrap2a decreases basal and ligand-induced Mc4r signaling, while *mrap2a* knockout in zebrafish decreases growth in the larval period. Additionally, Mrap2b reduces ligand-induced Mc4r signaling, and *mrap2b* knockout inhibits growth in adults [115].

Another series of studies involves the introduction of the *mc4r* knockout fish model. These studies provide further evidence of the essential role of Mc4r in regulating energy balance in bony fish [65,66,70]. In channel catfish, growth is consistently greater in Mc4r mutants than in WT at all life stages, both in ponds and tanks. A positive relationship between zygosity and growth is observed, with F1 homozygous/bi-allelic mutants reaching market size 30% more quickly than F1 heterozygotes. At the stocker stage (approximately 50 g), fish harboring homozygous *mc4r* mutations are 40% larger than control families [65]. Channel catfish with mutated *mc4r*, introduced through electroporation or microinjection, show a 38% and 20% increase in body weight, respectively, compared to the WT. Furthermore, mutated *mc4r* fish also demonstrate a lower feed conversion ratio (FCR, determined by the amount of feed consumed per unit of weight gained) than control fish [66]. In red crucian carp, *mc4r*^+/−^ fish generated using the CRISPR–Cas9 system show increased food intake, length, body weight, and body depth compared to control fish [70].

*However, mc3r*^+/−^ red crucian carp has slightly increased length and body depth, but has no effect on body weight compared to WT red crucian carp [70]. Similarly, *Mc3r*^−/−^ mice are not significantly overweight [19,20].

Across fish models, the strongest physiological evidence links reduced Mc4r signaling to increased feeding and/or enhanced growth, whereas Mc3r appears to exert more subtle effects on feed efficiency, growth shape, and metabolic partitioning (Table 3). However, increased feed intake alone is not equivalent to improved aquaculture performance. From a production perspective, the critical outcomes are growth rate under defined culture conditions, specific growth rate, feed conversion ratio, protein efficiency, body composition, and uniformity at market size. Future studies should therefore separate appetite-driven effects from true improvements in feed efficiency and growth quality, ideally using long-term experiments across developmental stages and commercially relevant rearing environments.

### 3.2. Glucose and Lipid Homeostasis

The neural MCRs have a direct and immediate impact on insulin sensitivity and glucose balance, independent of their effects on body weight and food intake [132,133,134,135,136,137,138,139]. ICV administration of MTII leads to a dose-dependent reduction in basal insulin release and enhances insulin sensitivity across different animal models, including those with genetic and diet-induced obesity [132,137]. The central administration of NDP-MSH causes a reduction in serum insulin, a response that is decreased by HS014 [139]. ICV administration of MTII or α-MSH significantly boosts insulin’s impact on both glucose production and uptake, while SHU9119 (an antagonist for MC3R and MC4R) exerts opposing effects [133,135]. Transgenic overexpression of α-MSH enhances glucose metabolism in models of both diet-induced and genetic obesity [140,141]. MC3R may also play a role in modulating insulin sensitivity [138].

The neural melanocortin system also has an important role in regulating lipid metabolism. AMP-activated protein kinase (AMPK) plays a central role in regulating fatty acid oxidation in skeletal muscle, and the ICV administration of MTII boosts AMPK activation in the skeletal muscle of mice fed a high-fat diet [142], indicating that the hypothalamic melanocortin system could be involved in the regulation of fatty acid mobilization [143]. Blocking MC4R or knocking out *Mc4r* significantly promotes triglyceride formation, fat accumulation, and lipid uptake in white adipose tissue. Conversely, MC4R activation in the CNS promotes lipid mobilization, regardless of food intake [144]. MC4R activation regulates dietary fat intake and leads to reduced fat consumption [145], while blocking the receptor with agouti, AgRP, or *Pomc* knockout promotes fat consumption [146,147,148].

Neural Mcr-mediated regulation of glucose and lipid homeostasis has also been studied in fish [70]. In red crucian carp, *mc4r*^+/−^ fish show elevated visceral fat mass compared to both *mc3r*^+/−^ and WT fish. Additionally, *mc3r*^+/−^ fish have a modest increase in visceral fat mass than control fish. These results indicate the significant involvement of fish Mc3r and Mc4r in lipid homeostasis. Furthermore, RNA-seq analysis of muscle and liver tissues identifies a considerable number of differentially expressed genes (DEGs) between *mc3r*^+/−^ and control, as well as *mc4r*^+/−^ and WT, primarily linked to glucose, lipid, and energy metabolism. These DEGs are mainly associated with pathways related to glycolysis/gluconeogenesis, fatty acid biosynthesis and metabolism, and steroid biosynthesis in the PPAR signaling pathway, the MAPK signaling pathway, and the Wnt signaling pathway. Notably, these pathways are predominantly implicated in lipid and glucose metabolism [70].

Current evidence suggests that fish Mc3r/Mc4r signaling influences not only feeding behavior but also nutrient partitioning, lipid storage, glucose metabolism, and broader metabolic remodeling (Table 3). This distinction is particularly important for aquaculture: faster growth accompanied by excessive visceral or hepatic lipid deposition may reduce product quality, compromise metabolic health, and increase susceptibility to environmental or nutritional stress. Future work should integrate growth measurements with hepatic lipid accumulation, muscle proximate composition, glucose and insulin-related endpoints, transcriptomic or metabolomic signatures, and long-term welfare indicators. Such integrated phenotyping will be essential for determining whether manipulation of neural Mcr signaling improves productive efficiency or merely shifts energy balance toward greater intake and lipid deposition.

### 3.3. Reproduction and Sexual Function

Leptin’s role in regulating both energy homeostasis and reproduction is well established. Neural MCRs act as the intermediary for leptin’s impact on energy regulation [149,150]. Furthermore, numerous studies propose that neural MCRs also contribute to leptin’s influence on reproductive function. ICV administration of AgRP increases follicle-stimulating hormone (FSH) and luteinizing hormone (LH) levels in rats and stimulates the release of gonadotropin-releasing hormone (GnRH) from hypothalamic explants, but does not directly impact LH release from the pituitary gland [151]. NDP-MSH stimulates GnRH secretion in hypothalamic GT1-1 cells (endogenously expressing *Mc4r*) [152,153]. MC4R antagonists diminish the prolactin (PRL) and LH surges under normal feeding conditions and effectively inhibit leptin-induced surges in starved rats [154]. AgRP eliminates PRL and LH surges in female rats, while the administration of anti-AgRP antiserum partially reverses the declines in PRL and LH surges [155]. These findings indicate the significance of the melanocortin system in hormonal surges among female rats. Further studies found that the preovulatory surge in PRL is mediated by the MC4R, not the MC3R [156]. In addition to hormone release, MC4R is also involved in regulating penile erectile function [157,158]. MC4R agonists, such as MTII [159], bremelanotide [160], and PF-00446687 [161], have also been studied as potential therapeutics for erectile dysfunction and premenopausal hypoactive sexual desire disorder.

In fish, both *mc3r* and *mc4r* genes show high expression in the gonads, brain, and pituitary glands of various fish species, suggesting that Mc3r/Mc4r may be involved in regulation of reproductive function (Table 1 and Table 2 and Figure 2). In larger male *Xiphophorus*, nonfunctional Y-linked *mc4r* copies serve as dominant-negative mutations, which delay the onset of puberty [48,162]. In spotted scat (*Scatophagus argus*), both THIQ (a selective MC4R agonist) and NDP-MSH increase the expression of follicle-stimulating hormone subunit beta (*fshb)*, luteinizing hormone beta (*lhb*), and *gnrh* genes in the hypothalamus in vitro at 3 and 6 h. Conversely, both nonselective (SHU9119) and selective (Ipsen 5i) antagonists of Mc4r decrease the expression of these genes. Similar results are observed in vivo when fish are intraperitoneally injected with THIQ and Ipsen 5i [54]. In black rockfish (*Sebastes schlegelii*), *mc4r* mRNA is detected in ovaries at various stages. Both α-MSH and β-MSH upregulate gonadotropin-inhibitory hormone (*gnih)* expression in the brain while reducing *sgnrh* and *cgnrh* expression. α-MSH decreases and β-MSH increases kisspeptin expression in the brain. These results imply that Mc4r could be involved in regulating GnRH secretion. Furthermore, α-MSH and β-MSH increase the expression of *cyp11*, *cyp19*, *3β-hsd*, and *star* in the ovaries [61]. The findings indicate that Mc4r may play a role in controlling the synthesis and secretion of steroid hormones in fish.

ASIP acts as an endogenous antagonist for MC4R. Transgenic zebrafish overexpressing *asip1* show higher egg production compared to WT females, though they spawn less often. These females also produce more fertilized eggs, but the hatching rate at 48 and 72 h is lower [114]. As far as we are aware, there are currently no reports on the involvement of fish Mc3r in reproduction.

The reproductive functions of fish neural Mcrs remain less well defined than their roles in feeding and growth, but the available evidence argues against treating Mcr inhibition as a growth-specific intervention (Table 3). Given the tight coupling between energy balance and reproduction in fish, altered Mc3r/Mc4r signaling may affect puberty timing, gonadotropin regulation, steroidogenesis, fecundity, gamete quality, and reproductive behavior. These considerations are especially relevant for broodstock management and for species in which growth, sexual maturation, and market value are closely linked. Future aquaculture-oriented studies should therefore include reproductive endpoints and sex-specific analyses when evaluating Mc3r/Mc4r-targeted breeding, genome editing, or pharmacological strategies.

## 4. Pharmacology of Neural MCRs

### 4.1. Mammalian Neural MCRs

Mammalian MC3R and MC4R are activated by POMC-derived melanocortin peptides and inhibited by endogenous antagonists [163,164]. α-MSH and β-MSH activate MC3R and MC4R, whereas γ-MSH shows relative preference for MC3R. AgRP acts as an endogenous antagonist and inverse agonist at neural MCRs, reducing basal receptor activity and promoting positive energy balance [165,166,167,168,169]. ASIP mainly antagonizes MC1R and MC4R [170,171]. These ligand–receptor relationships provide the canonical pharmacological framework for interpreting neural melanocortin signaling.

Neural MCR function is regulated by the melanocortin-2 receptor accessory proteins (MRAPs), MRAP1 and MRAP2. Although MRAP1 is essential for MC2R trafficking and ACTH responsiveness, both MRAP1 and MRAP2 can modulate MC3R and MC4R cell-surface expression, ligand potency, ligand selectivity, and basal or agonist-induced signaling [172,173,174,175]. The direction and magnitude of these effects vary depending on receptor subtype, MRAP isoform, ligand, and cellular context. This context dependence is important when comparing mammalian and fish MCR pharmacology.

Mammalian MC4R displays constitutive activity [168], and altered basal signaling has been implicated in the functional consequences of some obesity-associated *MC4R* variants [14,176]. AgRP and several synthetic inverse agonists (including ML00253764, Ipsen 5i, and MCL0020) can reduce basal MC4R activity [169,177,178,179,180,181,182]. Neural MCRs also signal through pathways beyond the canonical Gs-cAMP axis. Ligands such as AgRP and several small-molecule or peptide ligands can show pathway-selective activity, including differential effects on Gs-cAMP, ERK1/2, AKT, Gi, calcium, or Kir7.1-related signaling [168,181,183,184,185,186,187,188,189,190]. Thus, mammalian studies establish three concepts that are especially relevant for fish Mcrs: receptor basal activity, modulation by MRAP proteins, and pathway-selective or biased signaling. The following section focuses on how these principles are conserved, modified, or diverge in fish neural Mcrs.

### 4.2. Fish Neural Mcrs

**Figure 6:**
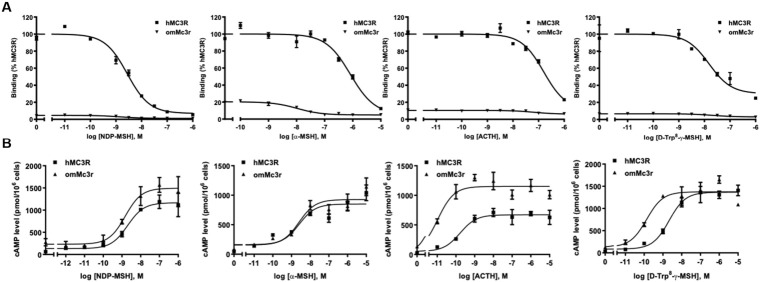
Pharmacology of rainbow trout Mc3r. (**A**) Ligand-binding properties of rainbow trout (om) omMc3r. HEK293T cells were transiently transfected with hMC3R or omMc3r plasmids, and the binding properties were measured 48 h later by displacing the binding of ^125^I-NDP-MSH using different concentrations of unlabeled NDP-MSH, α-MSH, ACTH (1–24), or D-Trp^8^-γ-MSH. Data are expressed as % of hMC3R binding ± range from duplicate measurements within one experiment. The curves are representative of at least three independent experiments. (**B**) Signaling properties of omMc3r. HEK293T cells were transiently transfected with omMc3r or hMC3R plasmids, and intracellular cAMP levels were measured by radioimmunoassay after stimulation with different concentrations of NDP-MSH, α-MSH, ACTH (1–24), or D-Trp^8^-γ-MSH. Data are means ± SEM from triplicate measurements within one experiment. All experiments were performed at least three times independently. These rainbow trout Mc3r data are summarized here as previously unpublished data; detailed methods followed those described previously for fish Mc3r pharmacological characterization [76].

The pharmacology of fish neural Mcrs has been studied in several species and shows several distinctive features. Fish Mc3r/Mc4r generally exhibit pharmacological properties that partially overlap with those of mammalian MC3R/MC4R, suggesting conservation of some ligand-recognition mechanisms (Table 4 and Table 5). Another interesting finding in fish Mc3r/Mc4r is that many fish Mc3r/Mc4r characterized to date show relatively strong ACTH responsiveness, although the rank order of ligand affinity and efficacy varies by species, receptor subtype, and assay system (Table 4 and Table 5 and Figure 6). These comparative pharmacological findings support the hypothesis that ACTH responsiveness may represent an ancient feature of MCR signaling. However, they do not establish ACTH as the original ligand for all MCRs, and this possibility requires broader phylogenetic and functional testing [44,53,55,56,57,58,62,64,75,76,81,191].

**Figure 7:**
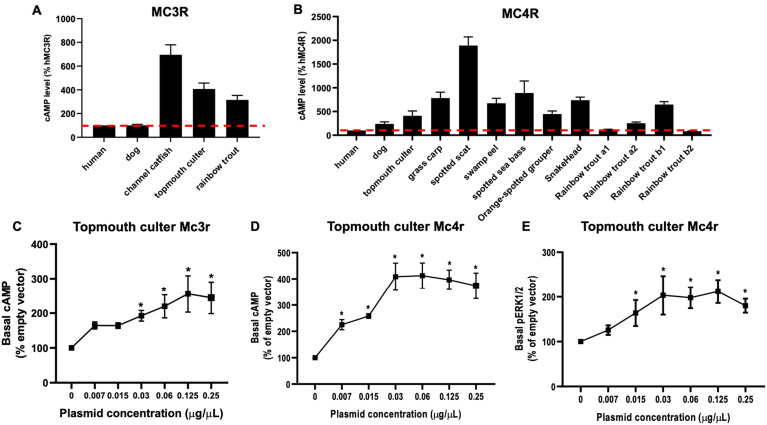
Basal activities of fish neural Mcrs. (**A**) Basal activities of MC3R; (**B**) basal activities of MC4R. Data are from our previous studies (Table 4 and Table 5). (**C**) Constitutive activities of fish Mc3r. Data were reproduced with permission from Ref. [76]. (**D**,**E**) Constitutive activities of fish Mc4r. Data were reproduced with permission from Ref. [62]. Red dashed line in (**A**,**B**) indicates 100% of human receptor as control. * *p* < 0.05.

The high basal activities in Gs-cAMP signaling are observed in all fish Mc4r studied, including zebrafish [192], Mexican cavefish [52], grass carp (*Ctenopharyngodon idella*) [55], spotted scat (*Scatophagus argus*) [53], orange-spotted grouper (*Epinephelus coioides*) [58], swamp eel (*Monopterus albus*) [56], spotted sea bass (*Lateolabrax maculatus*) [57], topmouth culter [62], swordtail (*Xiphophorus*) [193], Nile tilapia [60], snakehead (*Channa argus*) [64], and rainbow trout [69], and Mc3r studied by our lab and other groups, such as zebrafish [85], channel catfish [75], topmouth culter [76], and rainbow trout (Table 6 and Figure 7). High basal activity has also been observed in fish Mc1r [194], but not in fish Mc5rs [195,196]. Additionally, constitutive activation of Gs-cAMP signaling has been reported in lamprey MCa and MCb [197], as well as in chicken MC4R [198] and MC3R [199] and frog MC3R and MC4R [200]. Constitutive activity in ERK1/2 signaling exclusively is found in certain fish Mc4rs, including topmouth culter [62], spotted scat [182], grass carp [182], and rainbow trout [69], but not others. The significance of constitutive activity in teleost Mc3r/Mc4r requires further investigation.

Although the TMDs of MCRs are conserved across teleosts and mammals, there is reduced similarity in the N termini and ECLs of fish Mc4rs compared to hMC4R. It is well established that N termini and ELs play vital roles in modulating basal activities in hMC4R [176,201], thyroid-stimulating hormone receptor [202,203], and luteinizing hormone receptor [204]. In hMC4R, the conserved “HLWNRS” motif in N termini is suggested to contribute to basal activities [176,201]. However, a recent study found that this motif might not be involved in regulating basal activity [205]. N termini have only a partial role in regulating basal activity, and the second ICL2 has important roles in modulation of constitutive activity [205]. More research is required to determine if these domains are involved in the constitutive activation of fish Mc4r and to investigate whether increased basal activities of Mc4r are more widespread in teleosts.

AgRP (83–132) functions as a selective antagonist on neural MCRs in mammals [165,166]. Subsequent studies indicate that AgRP acts as an inverse agonist, reducing the constitutive activity of neural MCRs [167,168,169]. Similar results are also found in fish neural Mcrs. AgRP reduces the basal activity of Mc3rs in zebrafish [85] and channel catfish [75] and Mc4rs in sea bass (*Dicentrarchus labrax*) [47], spotted scat [182], grass carp [182], Nile tilapia [60], and rainbow trout [69]. AgRP acts as an antagonist, blocking ACTH-induced cAMP signaling in Nile tilapia [60].

Fish possess four endogenous antagonists: Agrp1, Agrp2, Asip1, and Asip2 [206]. Of these, Agrp1 shows high similarity to tetrapod AgRP at its C-terminal region (amino acids 83–132), being defined by ten conserved cysteine residues forming five disulfide bonds and a highly conserved Arg–Phe–Phe (RFF) motif. This motif is vital for ensuring the structural stability and functional efficacy of AgRP [39,207]. Consequently, the C-terminal fragment of human AgRP shows inverse agonist activity on fish Mc4rs, implying that AgRP-driven inverse agonism on MC4R has been evolutionarily conserved among vertebrate species. Agrp2, a homolog missing in the tetrapod [208], is expressed in the pineal gland and the optic nerve connecting the pituitary gland [192,209]. It is involved in background adaptation, stripe pattern formation, and neuroendocrine regulation of cortisol production [192,209,210]. Investigating the inverse agonism and antagonism of teleost Mc3r/Mc4r could yield important insights for optimizing feed intake and improving growth performance in aquaculture.

It is worth mentioning that notable pharmacological differences have been observed between mammalian and teleost MC4Rs in their response to small-molecule ligands, including THIQ, Ipsen 5i, MCL0020, and ML00253764. MCL0020 acts as a neutral antagonist for hMC4R [177,179,182]. MCL0020 competes with NDP-MSH for binding in humans, spotted scat (saMc4r), and grass carp (ciMc4r) MC4Rs [182]. In rainbow trout, the four Mc4r isoforms exhibit distinct pharmacological responses to MCL0020. Specifically, MCL0020 only binds to omMc4rb1, and not to omMc4ra1, omMc4ra2, or omMc4rb2 [69]. For signaling, MCL0020 inhibits NDP-MSH-stimulated cAMP production and reduces the basal activity of ciMc4r. However, it does not influence either basal or ligand-induced cAMP levels in saMc4r [182]. Additionally, MCL0020 does not impact cAMP signaling of the four omMc4rs [69].

Both ML00253764 and Ipsen 5i bind orthosterically to hMC4R and have been found to reduce the basal activity of both WT and constitutively active mutant hMC4Rs [56,169,178,180,181,182]. In fish, neither Ipsen 5i nor ML00253764 orthosterically bind to rice-field eel Mc4r (maMc4r), four trout Mc4rs, ciMc4r, and saMc4r, by displacing radiolabeled NDP-MSH, implying that the binding sites for these drugs are distinct between hMC4R and fish Mc4rs [56,69,182]. Furthermore, both compounds do not elicit significant effects on the cAMP levels of three omMc4rs (except for omMc4rb2) [69], saMc4r, and ciMc4r [182], while ML00253784 reduces the basal cAMP levels in maMc4r [56] and increase cAMP generation in omMc4rb2 [69]. Ipsen 5i stimulates cAMP production in maMc4r [56].

THIQ binds orthosterically to hMC4R, but allosterically to fish Mc4r, including spotted scat [53], swamp eel [56], spotted sea bass [57], and topmouth culter [62], where THIQ does not displace radiolabeled NDP-MSH but stimulates cAMP generation. These results indicate that THIQ functions as an allosteric agonist for teleost Mc4rs.

Biased signaling in fish Mc4rs has also been investigated [69,182]. Three small molecules (MCL0020, ML00253764, and Ipsen 5i) function as biased ligands for fish Mc4rs (grass carp and spotted scat) and hMC4R, demonstrating a preference for activating ERK1/2 signaling [181,182,211]. In rainbow trout, they specifically trigger ERK1/2 signaling in omMc4rb1, whereas they do not elicit a response in the other three omMc4r isoforms. Additionally, ML00253764 activates cAMP signaling without influencing ERK1/2 activation in omMc4rb2 [69]. These results suggest that MCL0020, ML00253764, and Ipsen 5i act as biased allosteric ligands for omMc4rb1, preferentially stimulating ERK1/2 activation, and ML00253764 functions as an allosteric agonist for omMc4rb2, selectively activating cAMP signaling. In the Gs-cAMP pathway, AgRP acts as an inverse agonist at constitutively active mutant and WT hMC4R [181,211], as well as fish Mc3rs [75,85] and Mc4rs [47,69,182,193]. In the ERK1/2 pathway, AgRP acts as a biased agonist, stimulating the ERK1/2 activation at human and fish MC4Rs, including spotted scat [182], grass carp [182], and rainbow trout (omMc4ra1 and omMc4rb1) [69].

**Table 6:** Pharmacological properties of Mrap2-regulated neural Mcrs in fish.

Species	Mc4r/Mraps	Trafficking	Binding Affinity	Potency	Efficacy	Basal Activity	Reference
Zebrafish	Mrap2a	no effect	no effect on α-MSH	no effect for α-MSH	decrease (α-MSH)	decrease	[115]
	Mrap2b	increase	no effect on α-MSH	no effect for α-MSH	no effect (α-MSH)	decrease	
	Mrap2a	NA	NA	increase for ACTH and no effect for α-MSH	no effect (α-MSH/ ACTH)	no effect	[212,213]
	Mrap2b	NA	NA	increase for ACTH and no effect for α-MSH	no effect (α-MSH/ ACTH)	no effect	
Orange-spotted grouper	Mrap2	NA	NA	NA	decrease (α-MSH)	decrease	[58]
Nile tilapia	Mrap2	decrease	NA	increase for α-MSH and no effect for ACTH	decrease (α-MSH/ ACTH)	decrease	[59]
	Mrap2b	NA	NA	increase for ACTH and no effect for α/β-MSH	increase (α-/β-MSH and ACTH)	decrease	[60]
Topmouth culter	Mrap2a	increase	increase in ACTH and no effect for α-MSH	no effect for α-MSH and ACTH	Decrease (α-MSH/ ACTH)	decrease	[62]
	Mrap2b	increase	increase in ACTH and no effect for α-MSH	no effect for α-MSH and ACTH	no effect (α-MSH/ ACTH)	decrease	
Swordtails	Mrap2	NA	NA	increase for NDP-MSH	increase (NDP-MSH)	increase	[193]
Snakehead	Mrap2	no effect	increase in ACTH and no effect for α-MSH	no effect for α-MSH and ACTH	decrease (α-MSH/ ACTH)	decrease	[64]
Rainbow trout	Mrap2a	NA	NA	increase for ACTH and NDP-MSH	increase (ACTH and NDP-MSH)	decrease	[214]
Elephant shark	Mrap2	NA	NA	no effect for ACTH	decrease (ACTH)	NA	[73]
	**Mc3r/Mraps**						
Zebrafish	Mrap2a	NA	NA	no effect for α-MSH	no effect (α-MSH)	no effect	[115]
	Mrap2b	NA	NA	no effect for α-MSH	no effect (α-MSH)	no effect	
Channel catfish	Mrap2	NA	NA	no effect for α-MSH	decrease (α-MSH/ ACTH)	decrease	[75]
Topmouth culter	Mrap2a	decrease	no effect on α-MSH and ACTH	no effect for α-MSH or ACTH	decrease (α-MSH)	decrease	[76]
	Mrap2b	no effect	no effect on α-MSH and ACTH	no effect for α-MSH or ACTH	no effect (α-MSH)	decrease	
Rainbow trout	Mrap2a	NA	NA	decrease for ACTH and NDP-MSH)	decrease (ACTH and NDP-MSH)	decrease	[214]
Elephant shark	Mrap2	NA	NA	no effect for ACTH	no effect (ACTH)	NA	[73]

NA: not available. Signaling data are for Gs-cAMP only.

Mrap-regulated Mcr signaling has also been studied in fish. In zebrafish, *mrap2a*^−/−^ decrease fish length in the larval stage [115]. The effect of Mraps on Mc3r/Mc4r differs across fish species (Table 6). The two Mrap2s (Mrap2a and Mrap2b) found in zebrafish exert distinct effects on Mc4r pharmacology. Mrap2a, present during the larval stage, acts as an antagonist by inhibiting Mc4r signaling through the blockade of ligand-receptor binding, resulting in decreased ligand-induced signaling. Although Mrap2a does not affect Mc4r trafficking, it does decrease basal activity in Gs-cAMP signaling. In contrast, Mrap2b, expressed later in development, enhances signaling by binding to Mc4r and increasing receptor sensitivity to the ligand. Furthermore, Mrap2b promotes an increase in Mc4r cell surface expression while decreasing basal activity [115]. Distinct effects on Mc4r by Mrap2a and Mrap2b have also been reported in topmouth culter. Both Mrap2 isoforms increase Mc4r cell surface expression and affinity to ACTH (but not to α-MSH), while decreasing basal cAMP signaling. They have no effect on potency to ACTH and α-MSH. However, Mrap2a suppresses signaling induced by both α-MSH and ACTH, while Mrap2b does not [62].

In orange-spotted grouper, Mrap2 reduces both basal and α-MSH-induced cAMP signaling, but enhances basal and α-MSH-mediated ERK1/2 activation at Mc4r [58]. In Nile tilapia, Mrap2 lowers Mc4r cell membrane expression, basal and α-MSH/ACTH-induced cAMP levels while enhancing potency specifically to α-MSH, but not to ACTH [59]; Mrap2b decreases basal cAMP level, whereas it increases ligand (α-MSH, β-MSH, and ACTH)-induced cAMP signaling, and potency to ACTH [60]. In swordtail, Mrap2 increases both the potency and efficacy of NDP-MSH and also enhances basal cAMP signaling at Mc4r [193]. In snakehead, Mrap2 has no effect on Mc4r trafficking or potency to ACTH and α-MSH. However, it increases affinity to ACTH (but not to α-MSH) and decreases basal and agonist (ACTH and α-MSH)-stimulated cAMP signaling [64]. In rainbow trout, Mrap2a increases both the potency and efficacy of ACTH and NDP-MSH, while inhibiting basal activity in cAMP signaling. However, it does not alter basal or agonist-induced ERK1/2 signaling [214].

Mrap2 also shows varying effects on Mc3r in various fish species, including zebrafish [115], topmouth culter [62], channel catfish [75], and rainbow trout [214]. In zebrafish, Mrap2s (Mrap2a and Mrap2b) do not influence the potency or efficacy of α-MSH or basal cAMP signaling at Mc3r [115]. In topmouth culter, Mrap2a reduces Mc3r expression on the cell membrane, as well as basal and maximal responses to both α-MSH and ACTH, while not affecting binding affinities and potencies. Conversely, Mrap2b only reduces basal cAMP level without impacting other aspects compared to Mrap2a [62]. In channel catfish, Mrap2 reduces basal and ligand (ACTH and α-MSH)-mediated cAMP levels, but does not impact ERK1/2 signaling [75]. In rainbow trout, Mrap2a decreases the potency and efficacy of ACTH and NDP-MSH, along with reducing basal activity in cAMP signaling. Regarding ERK1/2 signaling, Mrap2 increases basal ERK1/2 activity while decreasing agonist (ACTH and NDP-MSH)-induced ERK1/2 signaling [214].

In summary, Mrap2 can regulate Mcr trafficking, ligand binding, and signaling pathways, encompassing both ERK1/2 and Gs-cAMP, while also impacting ligand selectivity. These effects vary in a species-dependent manner in fish. Mrap2-regulated Mcrs have also been reported in cartilaginous fish [73]. In mammals, *MRAP2* is predominantly expressed in the CNS and is involved in regulating energy balance [215]. *Mrap2*^−/−^ mice develop severe obesity at an early age [216]. Recently, several studies have identified *MRAP2* mutations from obese patients [216,217,218,219], and impaired GPCR signaling by MRAP2 mutants may be mechanisms leading to obesity.

Fish neural Mcrs preserve important features of vertebrate neural MCR pharmacology, including responsiveness to POMC-derived peptides, but they also show striking divergence in constitutive activity, ACTH responsiveness, MRAP2 modulation, and small-molecule ligand behavior. These differences make direct extrapolation from mammalian MCR pharmacology unreliable. In particular, compounds classified as antagonists, inverse agonists, or biased ligands at mammalian receptors may display altered potency, efficacy, binding mode, or pathway selectivity at fish receptors. Species-specific pharmacological profiling should therefore precede any in vivo testing of Mcr-targeted compounds. Such profiling should include receptor expression, ligand binding where feasible, basal activity, Gs-cAMP signaling, ERK1/2 signaling, and Mrap2-dependent modulation so that “receptor inhibition” is defined mechanistically rather than inferred from mammalian ligand classification.

## 5. Fish mc4r Mutations

The first human *MC4R* frameshift mutations were identified in 1998 [23,24]. Since then, a variety of *MC4R* variants have been discovered. Individuals carrying *MC4R* mutations show characteristics such as hyperinsulinemia, hyperphagia, and increased bone mineral density [120,220,221,222,223]. To date, 679 mutations in *MC4R* have been reported [17,26,121,224]. On the Genomics 2 Proteins Portal (https://g2p.broadinstitute.org, accessed on 8 January 2025), 448 missense, 174 synonymous, 15 nonsense, 30 frameshift, 7 inframe indel, and 5 other mutations were catalogued. Mutations in *MC4R* that lead to early-onset morbid obesity emphasize the pivotal role of MC4R in maintaining human energy balance.

T6K and V81I MC3R were the first discovered, representing polymorphic variants in complete linkage disequilibrium [225,226]. Since then, researchers have identified 27 naturally occurring mutations in *MC3R* in individuals, spanning both obese and nonobese populations [16,27,227]. Among them, both I183N and I335S were exclusively discovered in obese subjects and have deficiencies in pharmacological properties [228,229,230,231,232,233]. These mutations are considered potential contributors to obesity or genetic factors that may increase susceptibility to excessive weight gain, especially adiposity [16,25,27,230,233,234,235,236]. Recently, we identified over 300 mutations in *MC3R* using data from the gnomAD v2.1.1 database (https://gnomad.broadinstitute.org/; accessed on 25 May 2024) [17,237]. Even more recently, on the Genomics 2 Proteins Portal (https://g2p.broadinstitute.org, accessed on 8 January 2025) [224], 469 missense, 186 synonymous, 14 nonsense, 22 frameshift, 11 inframe indel, and 6 other mutations (totaling 718 mutations based on the short isoform of 323 amino acids, lacking the first 37 amino acids of the previously widely used isoform) were catalogued (it should be noted that amino-acid numbering in older literature using the longer isoform should be decreased by 37 to be consistent with the shorter isoform, starting with the downstream methionine, P41968). These findings offer additional evidence of its involvement in regulating energy balance.

Biased mutants are receptor variants that, upon stimulation by endogenous ligands, selectively adopt specific active conformations, leading to distinct signaling outcomes across various signaling pathways [238]. Recently, there has been an increasing number of biased mutants identified within neural MCRs [185,239]. We also found biased signaling in 25 naturally occurring mutant MC4Rs, where these variants selectively activate either Gs-cAMP or ERK1/2 signaling in response to ligands [187]. Furthermore, mutant MC3Rs, whether naturally occurring or artificially generated, also demonstrate bias in ERK1/2 and Gs-cAMP signaling pathways [211,236,240,241].

A mutant *mc4r* allele was first reported in platyfish [48]. This fish offers a valuable case study on the genetic regulation of puberty onset in both sexes, male body size and reproductive success, and female fecundity. Interestingly, males reaching sexual maturity earlier tend to be smaller in adult size compared to those maturing later [242]. There are two mutant alleles in platyfish: B1 (lack two cysteine residues in C terminus) and B2 (lacks the CC motif and has an additional four-base deletion, resulting in a frameshift and elongated protein in the C terminus). This CC motif is highly conserved in vertebrate MC4R, marking the end of helix VIII in GPCR [243]. Functional studies found that these two mutants show defects in cAMP signaling and exhibit dominant negative effects on the WT receptor [48]. The dominant negative effects seen in these alleles differ from human MC4R variants, where mutant receptors retained intracellularly generally do not display such effects [14].

Several mutations in *mc4r* have also been reported in Mexican cavefish [52]. Tabin and his colleagues identified three missense mutations, occurring in conserved residues: M259T in TMD6, V162I in TMD4, and G145S in the second ICL, from surface and Tinaja cavefish. Subsequent studies demonstrated that the Mc4r variant has reduced basal and NDP-MSH-induced cAMP signaling. In vivo studies further confirm that the mutant allele (G145S) contributes to increased appetite, growth, and resistance to starvation [52]. The A154D variant in hMC4R has been linked to obesity [244]. Further studies on A154D hMC4R showed that this mutant also exhibits defects in NDP-MSH-induced ERK1/2 signaling [187]. These findings strongly indicate that impaired Mc4r signaling in cavefish is likely responsible for their obesity and hyperphagia phenotype, which represents a thrifty genotype advantageous in evolution.

Male body size is directly linked to the number of nonfunctional mc4r B alleles present [48]. In *Xiphophorus maculatus*, the *mc4r* gene has experienced a notable increase in copy number, reaching as many as 10 copies, along with a range of mutations, including promoter region variants, in-frame insertions/deletions, and both missense and nonsense mutations in the coding sequence. Functional receptor characterization revealed significant divergence in pharmacology among mutant receptors, including constitutive activity, ligand binding and hormone-stimulated signaling [245].

As far as the authors are aware, there have been no reports of mutations in fish *mc3r* to date.

Naturally occurring and engineered *mc4r* variants provide compelling evidence that attenuated Mc4r signaling can contribute to increased body size or enhanced growth in fish. Nevertheless, the phenotype associated with a given variant is unlikely to be determined by receptor activity alone. Zygosity, allele dosage, genetic background, developmental stage, nutritional environment, compensatory endocrine responses, and culture conditions may all influence the final growth outcome. Future studies should therefore pair genotype–phenotype association with receptor pharmacology, allele dosage analysis, body-composition measurements, reproductive assessment, and long-term performance testing before *mc4r* variants are adopted as markers for genomic selection or as targets for genome editing.

## 6. Intracellular Signaling Pathways of Neural MCRs

The conventional signaling pathway for neural MCRs involves their coupling with the stimulatory heterotrimeric G protein (Gs). Upon activation, neural MCRs stimulate adenylyl cyclase (AC), raising intracellular cAMP levels. This increase in cAMP activates protein kinase A (PKA), initiating downstream signaling processes. Gs protein-modulated signaling is the principal and most widely studied intracellular pathway for neural MCRs.

Besides coupling with Gs, MC4R also interacts with Gq and Gi proteins [246,247]. Gq protein activation leads to an increase in intracellular Ca^2+^ by stimulating phospholipase C β (PLCβ) and protein kinase C (PKC), whereas Gi protein decreases cAMP levels by inhibiting AC activity. In the GT1-1 murine hypothalamic cell line, which endogenously expresses MC4R, activation of MC4R causes a rise in intracellular Ca^2+^ through Gq/PLCβ signaling [248]. This pathway has also been observed in cells transfected with MC4R [249]. However, in GT1-7 cells, MC4R does not trigger Ca^2+^ mobilization [185]. Furthermore, MC4R also activates G_12/13_ [250].

MC4R is also involved in other signaling pathways that are independent of G proteins. Both in vivo and in vitro studies demonstrate that it is involved in the activation of the ERK1/2 pathway [153,251,252,253]. The mechanism underlying MC4R-mediated ERK1/2 signaling differs based on the ligand and cell type involved. In GT1-7 cells, MC4R-induced ERK1/2 activation in response to α-MSH is PKA-dependent [254]. MTII activates ERK1/2 signaling through a PKA-dependent pathway in MC4R-expressing neurons of the rat solitary nucleus [253]. In GT1-1 cells, NDP-MSH mediates ERK1/2 activation through the Ca^2+^/PKC pathway, whereas in HEK293 cells expressing MC4R, this signaling is mediated by Gi protein [153]. In CHO cells expressing MC4R, ERK1/2 signaling is activated via phosphatidylinositol 3-kinase (PI3K) [252]. Gain-of-function variants in MC4R show a signaling bias that leads to increased activation of ERK1/2 and enhanced recruitment of β-arrestin [255]. MC4R is involved in several other signaling pathways as well, including those involving protein kinase B (AKT), c-Jun N-terminal kinases (JNK), β-arrestin, 5′-AMP-activated protein kinase (AMPK), and potassium channel Kir7.1 [188,211,256,257,258].

Compared to MC4R, the investigation of MC3R-mediated intracellular signaling is relatively limited. Besides Gs signaling, MC3R was shown to interact with Gq and Gi proteins [259,260]. MC3R modulates intracellular Ca^2+^ release through an IP_3_-dependent mechanism, indicating the activation of the Gq pathway [259]. Additionally, MC3R functionally interacts with Gi proteins [260]. In HEK293 cells expressing MC3R, NDP-MSH induces ERK1/2 signaling via Gi rather than Ca^2+^, PKA, and PKC signaling [260], whereas AgRP induces ERK1/2 activation in a manner independent of PI3K and PKA [211]. MC3R has been shown to modulate the AKT pathway [211,261]. It also increases intracellular Ca^2+^ concentrations through both IP_3_-dependent and -independent mechanisms [259,262], inhibits AMPK signaling [211], and activates PKC pathway [263].

To date, extensive studies have shed light on the physiological roles of various intracellular signaling pathways activated by MC3R and MC4R. MC4R-activated Gs signaling is pivotal for eliciting anorexigenic signals in the hypothalamus, thereby promoting a negative energy balance. Mutant MC4Rs with constitutive activity have been discovered in obese individuals. This finding indicates that MC4R-mediated Gs signaling is not the sole pathway responsible for regulating energy balance [15]. Further studies have shown that MC4R mediates energy expenditure exclusively through the Gs signaling, with CNS-specific Gs deficiency causing a targeted defect in energy expenditure, while food intake remains unaffected [264,265].

Food intake mediated by MCRs is regulated by several other pathways. Deleting PVN-specific Gq results in hyperphagic obesity without changes in energy expenditure, implying that the control of food intake through MC4R is modulated by Gq signaling [246]. Activation of MC4R in PVH neurons reduces AMPK signaling, leading to the inhibition of food intake [256]. MC4R-mediated ERK1/2 signaling is believed to play a role in regulating energy balance by decreasing food intake [253,254]. In addition, the inhibition of food intake triggered by MC4R activation depends on its interaction with the closure of the potassium channel Kir7.1, leading to the depolarization of PVN neurons [188].

MC3R plays a key role in regulating energy balance by controlling circadian rhythms and affecting feed efficiency. However, the connection between its G protein-mediated intracellular signaling and physiological outcomes has not been fully studied. Furthermore, MC3R-regulated ERK1/2 signaling is thought to play a role in regulating feeding behaviors [125], as well as in anti-inflammatory effects [190] and mediating cell proliferation [260].

In fish, neural Mcrs demonstrate conserved intracellular signaling pathways, including the activation of ERK1/2 and Gs-cAMP signaling pathways [58,62,69,75]. Additionally, several studies have reported the ability of fish neural Mcrs to induce the NF-κB pathway [78,79,214]. In red crucian carp, RNA-seq analysis comparing liver and muscle tissues between *mc3r*^+/−^ and WT, as well as *mc4r*^+/−^ and WT, reveal differentially expressed genes predominantly enriched in pathways such as the PPAR, Wnt, and MAPK signaling pathways. These findings suggest that neural Mcrs may play a role in regulating these pathways [70].

Most functional studies of fish neural Mcrs have focused on Gs-cAMP signaling, reflecting its central role in canonical melanocortin receptor pharmacology. However, emerging evidence indicates that ERK1/2, NF-κB, and potentially other signaling pathways may also contribute to receptor function in fish. Importantly, a pathway that appears secondary in a heterologous cell system may be physiologically relevant in a particular tissue, developmental stage, or nutritional state. Future studies should therefore combine receptor-proximal pharmacology with endogenous tissue readouts, pathway-selective perturbation, and in vivo phenotyping. This integrated approach will be required to define which intracellular pathways mediate feeding, growth, metabolism, reproduction, immune regulation, and stress responses downstream of fish Mc3r/Mc4r.

## 7. Evolution of the Melanocortin System

Two melanocortin receptor-related sequences, MCa and MCb, have been identified in lampreys and provide useful information for understanding early vertebrate MCR evolution. However, the functional classification of these receptors should be interpreted cautiously, particularly for MCb, for which melanocortin peptide binding and activation have not been fully established [197,266]. Based on their gene structure and chromosome localization, vertebrate MCRs have been proposed to have diversified through ancestral duplication events. In this model, an MCb-related branch gave rise to MC3R and MC4R, whereas an MCa-related branch contributed to MC1R and the ancestral MC2R/MC5R lineage [267]. A local duplication of the ancestral MC2R/MC5R gene may have subsequently generated separate MC2R and MC5R genes. Current genomic and phylogenetic evidence supports a vertebrate-centered expansion of the MCR family, with well-supported orthologs in lampreys, cartilaginous fish, ray-finned fish, and tetrapods. Broader sampling of jawless vertebrates and non-vertebrate chordates will be needed to refine the timing of MCR emergence.

We recently examined five MCR-like receptors from urochordates and cephalochordates, specifically from *Ciona intestinalis*, *Styela clava*, *Branchiostoma belcheri*, and *Branchiostoma floridae*. Our phylogenetic analyses indicated a link between vertebrate MCRs and these receptors. However, these receptors lack several key residues that are crucial for MCR function in vertebrates. Subsequent studies revealed no specific binding or signaling (cAMP or ERK1/2 activation) in response to endogenous α-MSH or synthetic MC4R ligands. Interestingly, several of these receptors exhibited high constitutive activity in cAMP signaling, probably due to ligand-independent Gs coupling. These findings suggest that these receptors should not be regarded as functional orthologs of vertebrate MCRs, but rather as related ancient class A GPCRs with currently unidentified endogenous ligands [89].

Comparative genomic and phylogenetic studies support a model in which MCRs and POMC-derived ligands became functionally linked during early vertebrate evolution [268,269,270]. However, the precise timing and sequence of receptor–ligand co-diversification remain unresolved. POMC, an ancient gene that likely evolved from an ancestral opioid-coding gene after early vertebrate genome duplication events, is classified within the opioid/orphanin family, which also includes proorphanin, proenkephalin, and prodynorphin [271]. In tetrapod species, POMC consists of the C-terminal β-lipotropin, central ACTH, and N-terminal pro-c-MSH, with each region containing an MSH peptide defined by the HFRW core sequence. In sea lamprey, two POMC-related precursor genes, proopiocortin (POC) and proopiomelanotropin (POM), have been identified [272]. POC encodes β-endorphin and ACTH-related sequences, whereas POM contains MSH-core and opioid-related sequences [272]. These lamprey precursors provide important comparative information, but they should not be described simply as canonical vertebrate POMC orthologs. The timing of POMC’s origin remains uncertain, with questions surrounding whether POMC evolved prior to the divergence of lampreys and gnathostomes. Melanocortin-like peptide of *E. coli* (MECO-1) has been found to work through the MC1R to exert anti-inflammatory effect, despite low homology to α-MSH [273]. There are also reports of POMC-derived peptides in invertebrates [274]. Nevertheless, the relationship of these peptide-like signals to the vertebrate melanocortin system remains uncertain, and additional genomic and functional evidence is needed before they can be incorporated into a unified model of MCR–POMC evolution.

Fish provide a diverse and evolutionarily informative system for studying melanocortin system diversification. The melanocortin system in fish may have undergone various evolutionary processes. For instance, zebrafish possess six *mcr* genes, with two copies of *mc5r* [38,275]. Four *mc4r* genes have been identified in salmonids [63,69]. The presence of *mc3r* gene varies among fish species, as discussed above [76]. For the *pomc* gene, numerous fish species possess two or three *pomc* genes, with each gene encoding POMC containing three HFRW cores (except for γ-MSH), such as *pomca1*, *pomca2*, and *pomcb* in Nile tilapia and Atlantic salmon [60,63], and *pomca*/*pomcb* in rainbow trout [69]. Numerous fish species listed in the NCBI database are found to have two or three *pomc* genes (https://www.ncbi.nlm.nih.gov/gene/?term=pomc+fish, accessed on 25 May 2024). Interestingly, γ-MSH is absent in most teleost lineages but present in cartilaginous fish and tetrapods [276,277], suggesting lineage-specific retention or loss during vertebrate evolution. α-MSH is highly conserved across major vertebrate lineages, including cyclostomes and mammals [276,277].

MRAPs are now recognized as crucial components of the melanocortin system. In mammals, there is a single gene for both *MRAP2* and *MRAP1*. One evolutionary model proposes that MRAP2-like proteins preceded MRAP1, supported in part by the identification of an MRAP2-like protein in sea lamprey [278]. It has been proposed that an ancestral MRAP2-like gene duplicated during early vertebrate evolution, with one copy giving rise to MRAP1 before the diversification of ray-finned fish [276,279]. Previous research suggested that the *mrap1* gene is absent in amphibians and reptiles [278]. However, subsequent studies revealed that the *mrap1* gene is indeed present in amphibians and reptiles (https://www.ncbi.nlm.nih.gov/gene/?term=mrap+fish, accessed on 25 May 2024) [280,281].

In fish, *mrap1* was first reported in zebrafish [282]. Both MRAP1 and MRAP2 promote the transport of MC2R orthologs to the cell surface in teleosts and tetrapods [173,282]. Furthermore, the functional activation of these MC2R orthologs at physiological levels of ACTH requires their co-expression with MRAP1 [282,283,284,285,286]. Additionally, Mrap1 also plays a crucial role in modulating pharmacology of other Mcrs beyond Mc2r in fish [285,287,288,289,290].

To date, the *mrap1* gene has been reported only in a few teleost species, including zebrafish, rainbow trout, fugu, and spotted gar [276,278], as well as in cartilaginous fish [286,287,288,289,290,291,292]. According to data from the NCBI, the *mrap1* gene is present in numerous teleosts (https://www.ncbi.nlm.nih.gov/gene/?term=mrap+fish, accessed on 25 May 2024). Relatively low conservation in the MRAP1 protein is observed. For example, there is only 64% similarity between human and mouse MRAP1, and less than 50% identity between mammalian and fish MRAP1 [175].

Many fish species possess two isoforms of Mrap2, known as Mrap2a and Mrap2b (https://www.ncbi.nlm.nih.gov/gene/?term=mrap2a+Orthologs and https://www.ncbi.nlm.nih.gov/gene/?term=mrap2b+Orthologs, accessed on 25 May 2024). In zebrafish and culter, the two forms of Mrap2 exert different effects on the pharmacology of Mc3r/Mc4r [62,76,115]. Overall, data suggest that the melanocortin system may have undergone diverse evolutionary processes in fish. Further investigation is needed to explore this topic.

Fish offer a uniquely informative comparative system for studying melanocortin system evolution, retaining ancient vertebrate features while also exhibiting lineage-specific gene loss, gene duplication, ligand diversification, and receptor pharmacological divergence. Evolutionary conclusions, however, should be framed cautiously and should distinguish sequence homology and genomic synteny from ligand binding, receptor activation, and physiological function. Comparative pharmacology supports the hypothesis that ACTH responsiveness may represent an ancient feature of MCR signaling, but it should not be taken as definitive evidence that ACTH was the original ligand for all MCRs. Broader sampling of jawless vertebrates, cartilaginous fish, basal ray-finned fish, and diverse teleost lineages will be required to resolve when neural Mcr functions, constitutive activity, MRAP modulation, and ACTH responsiveness emerged during vertebrate evolution.

## 8. Strategies to Target Melanocortin System and Application in Aquaculture

**Figure 8:**
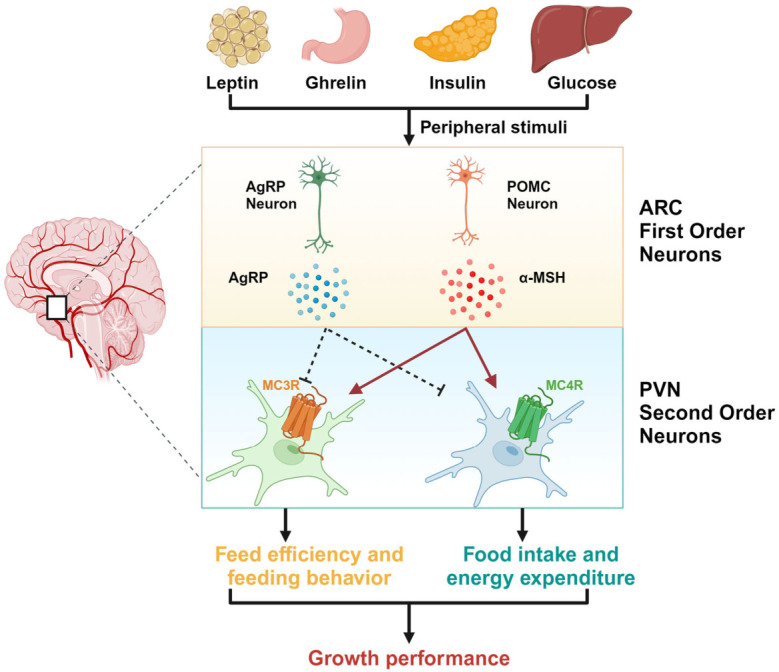
Conceptual model of hypothalamic melanocortin signaling and its potential relevance to fish growth regulation. POMC- and AgRP-expressing neurons sense peripheral metabolic signals and regulate downstream neurons expressing neural MCRs. In mammals, this ARC-based POMC/AgRP–MC3R/MC4R circuit is well established as a central regulator of appetite, energy expenditure, and metabolic homeostasis. In fish, Pomc, Agrp, Mc3r, and Mc4r have been identified and linked to feeding, growth, lipid/glucose metabolism, and reproduction; however, the organization of hypothalamic nuclei, the precise neuronal projections, receptor-expressing cell types, and ligand–receptor interactions may differ among species and remain incompletely resolved. Therefore, this figure should be viewed as a conceptual framework rather than a fully established fish neural circuit. Solid arrows indicate stimulatory signaling or activation, whereas dashed lines indicate inhibitory regulation. Figure created with BioRender.com.

As illustrated conceptually in Figure 8, the central melanocortin system links peripheral metabolic signals to hypothalamic control of appetite, energy balance, and growth. In mammals, leptin, ghrelin, insulin, and other metabolic cues are integrated by hypothalamic POMC and AgRP neurons, which regulate downstream MC3R- and MC4R-expressing neurons through POMC-derived agonists and AgRP-mediated antagonism or inverse agonism. In fish, homologous components of this system, including pomc, agrp, mc3r, and mc4r, have been identified, and pharmacological, genetic, and expression studies support roles in feeding, growth, lipid/glucose metabolism, and reproduction. However, the precise organization of fish hypothalamic nuclei, POMC/AgRP neuronal projections, receptor-expressing cell populations, and ligand systems differs among species and remains less completely resolved than in mammals. Therefore, this figure is intended as a conceptual framework for understanding how neural Mcr signaling may influence fish growth and aquaculture traits, rather than as a fully established circuit map for all fish species.

### 8.1. Development of Small-Molecule Compounds for Fish Mcrs

Teleost neural Mcrs show high basal activities in cAMP signaling (Figure 7). Decreased constitutive activity in hMC4R mutants is associated with obesity pathogenesis. In aquaculture, fish with reduced constitutive activity of Mcrs may show higher feed efficiency, lower metabolic rates, and faster growth, but these outcomes require species-specific and long-term validation. Additionally, inverse agonists, particularly small-molecule compounds, which can be added to diets, could be explored as tools to reduce constitutive activity in selected teleost Mcrs. Furthermore, antagonists blocking teleost Mcr signaling might also enhance fish growth. Therefore, inverse agonists and neutral antagonists targeting fish Mcrs (especially small-molecule compounds) may represent a potential strategy for modulating feeding and growth-related traits in aquaculture, provided that efficacy, residue risk, environmental fate, and regulatory feasibility are evaluated. As previously discussed, the distinct pharmacological profiles of small-molecule compounds on human and teleost MC4Rs emphasize the importance of conducting pharmacological studies on Mc4r of the intended species before any field trials in fish. This is particularly critical when considering the potential use of small-molecule ligands developed for mammalian MCRs.

**Figure 9:**
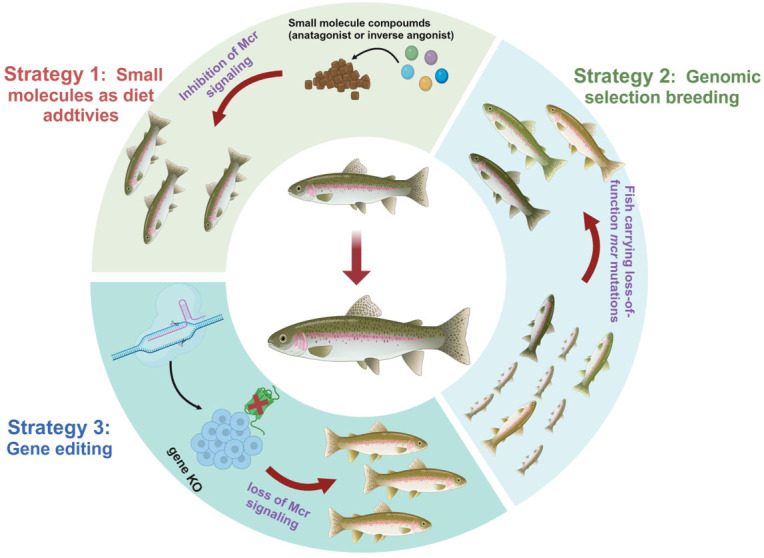
Strategies targeting Mcrs in aquaculture. The development of inverse agonists (which bind to receptors and reduce their basal activity) and neutral antagonists (which bind to receptors without activating them or reducing basal activity, but block agonist activation of the receptor) represents a novel feeding strategy in aquaculture, particularly through the use of small-molecule compounds as dietary additives (Strategy 1). Inhibiting Mcr signaling in fish can significantly promote growth. *mc3r* and *mc4r* may serve as candidate molecular markers for genomic selection of growth-related traits, pending species-specific validation. Fish with specific Mcr variants, such as loss-of-function mutations, can be selected to achieve desired growth traits (Strategy 2). Furthermore, utilizing CRISPR-Cas9 and other gene editing technologies to modify *mc4r* gene offers significant potential for application in aquaculture (Strategy 3). Figure created with BioRender.com.

Based on this approach, we propose the following methods (Figure 9): 1. utilizing small-molecule libraries to identify potential drugs targeting fish Mc3r/Mc4r; 2. employing tools such as AI to optimize existing or design new small-molecule drugs targeting fish Mc3r/Mc4r based on their structural characteristics; 3. validating the pharmacological characteristics of small-molecule drugs at the in vitro level; and 4. conducting in vivo tests to further evaluate the efficacy and safety of the identified compounds. Research efforts should prioritize the identification of exogenous low-cost inverse agonists that decrease the high constitutive activities of fish Mc3r/Mc4r or antagonists.

### 8.2. Genomic Selection Breeding Targeting Fish Mcrs

Genomic selection breeding of production traits holds significant potential for increasing efficiency and reducing the environmental footprint of aquaculture, with notable progress achieved recently [293,294]. As previously discussed, the melanocortin system plays a crucial role in fish growth. Therefore, genes related to the melanocortin system, particularly *mc4r* and possibly *mc3r*, may serve as candidate molecular markers for genomic selection of growth-related traits, pending species-specific validation (Figure 9).

Building upon previous studies, we propose a potential model for implementing the melanocortin system in aquaculture practices. Firstly, causative or functional variants identified in *mc3r* and/or *mc4r* through sequencing are associated with growth traits. Following sequencing, in vitro experiments are conducted to investigate the pharmacology and functional assays to evaluate the identified variants. Subsequently, the association between the phenotype and pharmacology of fish carrying the variant is examined. The fish harboring specific variants are selected to obtain desired growth traits.

### 8.3. Genome Editing Targeting Fish Mcrs

Gene- and genome-editing technologies, such as CRISPR–Cas9, TALENs, and ZFNs, provide powerful tools for advancing genetic advancements. The CRISPR–Cas9 system is distinguished by its efficiency, cost-effectiveness, and accuracy in gene editing, making it an increasingly popular tool for genome editing in aquaculture species (Figure 9). It has been applied to accurately modify genes, explore gene functions, and foster desired traits in more than 20 aquaculture species. These traits include those related to growth, sex/reproduction, pigmentation, fatty acid profiles, diseases, immunity, aquatic toxicity, and meiosis [295,296,297].

CRISPR–Cas9-edited *mc3r*/*mc4r* has also been reported in fish, including channel catfish and red crucian carp [65,66,70]. In these fish, homozygous or heterozygous knockout of *mc3r*/*mc4r* leads to improved growth performance. Furthermore, mutated Mc4r fish also demonstrate a lower FCR in channel catfish [66], and higher food intake in red crucian carp [70]. These findings support *mc4r* as a candidate marker and potential genome-editing target for growth-related traits in selected aquaculture species. However, application to other species will require species-specific validation of growth, feed efficiency, body composition, reproductive performance, welfare, and biosafety endpoints.

### 8.4. Targeting Fish Mrap2

MRAP2-regulated MCR signaling demonstrates dose dependence across vertebrates, including fish [58,59,60,62,64,75,76,115,175,194,195,196,197,199,200,214,275,280,298]. The *MRAP2* gene emerges as a novel candidate associated with monogenic obesity [218]. In aquaculture, Mrap2-regulated Mcr signaling in a dose-dependent manner provides an endogenous modulator for mediating Mcr signaling. Therefore, targeting Mrap2 to modulate Mc3r and Mc4r could potentially offer a novel approach to enhance growth in aquaculture.

### 8.5. Challenges and Biosafety Considerations for Aquaculture Application

Although targeting fish Mc3r/Mc4r signaling represents a promising strategy to improve growth-related traits, caution needs to be applied in its application to aquaculture. First, the pharmacology and physiological roles of neural Mcrs are species-specific. A ligand or genetic intervention that reduces Mc4r signaling in one species may not produce the same effect in another species, particularly given the diversity of receptor paralogues, Mrap modulation, basal activity, and ligand responsiveness among teleosts (Table 3, Table 4, Table 5 and Table 6). Therefore, species-specific receptor characterization and in vivo validation are essential before any practical application.

Second, growth promotion should not be evaluated solely by increased feed intake or body weight. Long-term inhibition of Mcr signaling may affect feed conversion ratio, feeding rhythm, hepatic or visceral lipid deposition, muscle quality, stress tolerance, immune competence, reproductive capacity, and fish welfare. These outcomes are particularly important: excessive lipid accumulation or altered reproductive maturation may reduce product quality or compromise broodstock performance [48,52,65,66,70,245]. Future studies should therefore assess specific growth rate, feed conversion ratio, protein efficiency, body composition, liver health, reproductive endpoints, stress responsiveness, and disease resistance under realistic culture conditions.

Third, the use of small-molecule inverse agonists or antagonists as feed additives raises additional biosafety and regulatory issues. Candidate compounds would require evaluation of absorption, metabolism, tissue residues, withdrawal periods, environmental release, and potential effects on non-target aquatic organisms or consumers. For genome editing or genomic selection strategies targeting *mc3r*/*mc4r*, ecological risk, genetic containment, animal welfare, and consumer acceptance should also be considered [295,296,297]. Although manipulation of fish neural Mcr signaling has considerable translational potential, its aquaculture application should proceed through a staged evaluation framework that balances productive benefit against biological trade-offs, biosafety, and regulatory feasibility, including receptor pharmacology in the target species, controlled feeding trials, long-term safety and welfare analyses, residue and environmental assessment, and field validation under commercial culture conditions.

## 9. Conclusions and Future Directions

Significant advancements have occurred in mammalian MC3R/MC4R research since their cloning just over three decades ago. Various methods, such as anatomical localization, pharmacological interventions, gene targeting, transgenes, and the development of small-molecule agonists and antagonists, have revealed multiple functions, including roles in cardiovascular function, energy homeostasis, sexual and reproductive function, cachexia, glucose and lipid homeostasis, pain perception, drug addiction, and mood. Due to their important roles and significant advancements, Mc3r/Mc4r have also been extensively studied in fish, demonstrating some conserved functions, including energy homeostasis, lipid and glucose metabolism, reproduction, and sexual function. However, there are significant differences observed in tissue expression and pharmacology between mammals and fish. In fish, the wider expression of *mc3r*/*mc4r* may suggest that these receptors have more diverse functions. However, research in this area in fish is currently lagging and requires further investigation.

Leveraging the latest research findings in the field of human medicine on MCRs can accelerate research advancements in aquaculture animals, particularly in the development of small-molecule drugs. However, noticeable differences in pharmacology have been observed in small-molecule compounds developed based on mammals when applied to fish Mcrs. Although targeting antagonism and inverse agonism of fish Mc3r/Mc4r could offer a potential strategy for boosting feed intake and growth in aquaculture, it is crucial to carefully evaluate the use of synthetic ligands, particularly small molecules designed for mammalian MCRs. It is essential to conduct a comprehensive examination of the Mc3r/Mc4r pharmacology in the target species before advancing to field trials. Furthermore, utilizing drug libraries and AI tools (such as AlphaFold) to predict the structure of fish Mc3r/Mc4r, or optimizing small-molecule drugs targeting mammalian MCRs, can further advance the development of low-cost small-molecule drugs targeting fish Mcrs.

Genetic variation or genome editing involving *mc4r* has been associated with growth-related advantages in several fish models. This suggests that targeting Mc4r may be a promising direction for future developments in aquaculture.

Due to the highly diverse and evolutionarily significant nature of fish species, they are ideal subjects for studying the origin and evolution of specific biological functions in vertebrates. Comparing fish and mammalian GPCRs can help us to understand the evolutionary patterns of their functions from an evolutionary perspective. Four *mc4r* genes have been identified in salmonids. Additionally, the presence of the *mc3r* gene varies among fish species. Furthermore, g-MSH is absent in teleosts. These observations suggest that the melanocortin system may have undergone diverse evolutionary processes. Therefore, fish Mcr research may provide insightful perspectives for understanding evolution.

In conclusion, this review offers an encyclopedic overview and critical discussion of the existing knowledge regarding teleostean neural Mcrs. We hope that this review will help guide future research on fish neural Mcr biology and support the cautious translation of Mcr-targeted strategies into aquaculture applications.

## Figures and Tables

**Table 1:** Research progress on fish Mc4r.

Species	Gene Name	Year	Tissue Expression	Reference
**Teleosts**				
Zebrafish	*mc4r*	2002	muscle, eye, GI, brain, and ovary	[37,38,43]
Fugu	*mc4r*	2003	brain, head kidney, and gut	[38,43,44]
Goldfish	*mc4r*	2003	eye, dorsal skin, gill, spleen, ovary, and all brain regions	[40,45]
Rainbow trout	*mc4r*	2004	brain, head kidney, and gut	[46]
Sea bass	*mc4r*	2009	retina, brain, and pituitary gland, liver, fat tissue, testis, and white muscle	[47]
Swordtail fish	*mc4r A1*	2010	brain and eye	[48]
	*mc4r B1*		brain and eye	
	*mc4r B2*		brain and eye	
Snakeskin gourami	*mc4r*		brain, testis, gill, liver, head kidney, trunk kidney, intestine, muscle, ovary, and stomach.	[49]
Common carp	*mc4r*	2012	brain, testis, and eye, pituitary and heart	[50]
Ya-fish	*mc4r*	2013	brain, ovary, heart, liver, pituitary, eye, spleen, skin, red muscle and testis	[51]
Mexican cavefish	*mc4r*	2015	brain	[52]
Spotted scat	*mc4r*	2016	brain, pituitary, and gonads, kidney, heart, gill, muscle, spleen and intestine	[53,54]
Grass carp	*mc4r*	2017	brain and eye, muscle, heart, intestine, liver, gill, spleen, and kidney	[55]
Swamp eel	*mc4r*	2018	brain, gonad, kidney, intestine, heart, muscle, and liver	[56]
Spotted sea bass	*mc4r*	2019	brain, pituitary, liver	[57]
Orange-spotted grouper	*mc4r*	2019	brain, pituitary gland, gill, liver, stomach, testis, kidney and spleen	[58]
Nile tilapia	*mc4r*	2019	brain, kidney, liver, muscle, intestine and stomach	[59,60]
Black				
rockfish	*mc4r*	2020	brain, liver, ovary and stomach	[61]
Topmouth culter	*mc4r*	2020	brain, pituitary gland, liver, testis, and head kidney	[62]
Atlantic salmon	*mc4ra1*	2020	brain	[63]
	*mc4ra2*		brain	
	*mc4rb1*		brain	
	*mc4rb2*		brain	
Snakehead	*mc4r*	2021	brain, adipose, brain, eye, gill, gonad (ovary), heart, intestine, kidney, liver, muscle, and spleen	[64]
Channel catfish	*mc4r*	2022	NA	[65,66]
Gibel carp	*mc4r*	2022	brain, gonad, skin, liver, and intestine	[67]
Mandarin fish	*mc4r*	2023	brain, liver, spleen, heart, intestine, muscle and kidney	[68]
Rainbow trout	*mc4ra1*	2023	NA	[69]
	*mc4ra2*		NA	
	*mc4rb1*		NA	
	*mc4rb2*		NA	
Red crucian carp	*mc4r*	2023	brain, gonad, muscle, and pituitary	[70]
**Cartilaginous fish**				
Dogfish	*mc4r*	2003	brain	[71]
Red stingray	*mc4r*	2016	brain	[72]
Elephant shark	*mc4r*	2019	brain, pituitary, gill, spleen, kidney, and gonad	[73]

NA: not available. Detailed accession numbers and amino-acid lengths are provided in Supplementary Table S1.

**Table 2:** Research progress on fish Mc3r.

Species	Year	Tissue Expression	Reference
**Teleosts**			
Zebrafish	2003	embryos and adult	[38,43]
Wuchang bream	2019	NA	[74]
Channel catfish	2019	NA	[75]
Topmouth culter	2021	brain, testis, liver, head kidney, skin, and ovary	[76]
Cavefish	2021	brain, liver, kidneys, muscle, heart, and gonads	[77]
Rainbow trout	2022	brain, muscle, liver, intestine, gonad, stomach, spleen and kidney	[78]
Red crucian carp	2023	brain, spleen, testis, head kidney, and skin	[70]
Common carp	2023	brain, intestine, kidney, liver, and spleen, heart, and muscles	[79]
Grass carp	2023	brain, muscle, kidney, spleen, heart, intestine, and liver	[80]
**Cartilaginous fish**			
Dogfish	2004	brain and eye	[81]
Red stingray	2016	brain	[72]
Elephant shark	2019	brain, gill, atrium, kidney, intestine, ovary, uterus, rectal gland, and testis	[73]

NA: not available. Detailed accession numbers and amino-acid lengths are provided in Supplementary Table S2.

**Table 4:** Pharmacological properties of fish Mc4r.

Species	Gene	Binding Affinity	Activity (cAMP Signaling)	Reference
**Teleosts**				
zebrafish	*mc4r*	NDP-MSH > β-MSH > α-MSH > γ1-MSH	NA	[37,38,43]
fugu	*mc4r*	MTII > ACTH > NDP-MSH > HS024 > α-MSH > γ1-MSH > β-MSH	NA	[44]
goldfish	*mc4r*	NDP-MSH > HS024 > MTII > β-MSH > α-MSH > γ1-MSH	NA	[40,45]
rainbow trout	*mc4r*	SHU9119 > NDP-MSH > MTII > HS024 > ACTH > α-MSH > β-MSH > γ1-MSH	NA	[46]
sea bass	*mc4r*	NA	Diacetly-MSH > MTII > α-MSH > β-MSH > Deascety-MSH	[47]
spotted scat	*mc4r*	NDP-MSH > ACTH > α-MSH	NDP-MSH > ACTH > α-MSH	[53]
grass carp	*mc4r*	NDP-MSH > ACTH > α-MSH > β-MSH	NDP-MSH > ACTH > β-MSH > α-MSH	[55]
swamp eel	*mc4r*	NDP-MSH > ACTH > α-MSH > β-MSH	NDP-MSH > α-MSH > ACTH > β-MSH	[56]
spotted sea bass	*mc4r*	NDP-MSH > ACTH > α-MSH	NDP-MSH > ACTH > α-MSH	[57]
orange-spotted grouper	*mc4r*	NDP-MSH > ACTH > α-MSH	NDP-MSH > ACTH > α-MSH	[58]
Nile tilapia	*mc4r*	NA	ACTH > α-MSH	[59]
topmouth culter	*mc4r*	NDP-MSH > ACTH > α-MSH > β-MSH	NDP-MSH > α-MSH > ACTH > β-MSH	[62]
snakehead	*mc4r*	NDP-MSH > ACTH > β-MSH > α-MSH	NDP-MSH > ACTH > α-MSH > β-MSH	[64]
rainbow trout	*omMc4ra1*	ACTH > α-MSH	α-MSH > ACTH	[69]
rainbow trout	*omMc4ra2*	ACTH > α-MSH	α-MSH > ACTH	[69]
rainbow trout	*omMc4rb1*	ACTH > α-MSH	α-MSH > ACTH	[69]
rainbow trout	*omMc4rb2*	ACTH > α-MSH	ACTH > α-MSH	[69]
**Cartilaginous fish**				
spiny dogfish	*mc4r*	NDP > α-MSH > β-MSH > γ1-MSH	NA	[71]
red stingray	*mc4r*	NA	Des-Ac-α-MSH > ACTH > γ-MSH > β-MSH > δ-MSH	[72]

NA: not available.

**Table 5:** Pharmacological properties of fish Mc3r.

Species	Binding Affinity	Activity (cAMP Signaling)	Reference
**Teleosts**			
channel catfish	NDP-MSH > ACTH > α-MSH > β-MSH > D-Trp^8^-γ-MSH	NDP-MSH > β-MSH > ACTH > α-MSH > D-Trp^8^-γ-MSH	[75]
topmouth culter	NDP-MSH > ACTH > α-MSH	α-MSH > NDP-MSH > ACTH	[76]
cavefish	NA	ACTH > NDP-MSH > β-MSH > α-MSH	[77]
rainbow trout	NA	ACTH > NDP-MSH > α-MSH > β-MSH	[78]
	NDP-MSH > D-Trp^8^-γ-MSH > ACTH > α-MSH	ACTH> D-Trp^8^-γ-MSH > NDP-MSH > α-MSH	#
common carp	NA	α-MSH > ACTH > β-MSH > NDP-MSH	[79]
grass carp	NA	β-MSH > NDP-MSH > ACTH > α-MSH	[80]
**Cartilaginous fish**			
spiny dogfish	α-MSH > ACTH > γ-MSH1	NA	[81]
stingray	NA	Des-Ac-α-MSH = ACTH = γ-MSH > β-MSH > δ-MSH	[72]

NA: not available; # unpublished data summarized in this review; see Figure 6.

## Data Availability

Most data summarized in this review were extracted from previously published studies cited in the text, tables, and figure legends. The rainbow trout Mc3r pharmacological data summarized in Figure 6 and Table 5 are presented here as unpublished data and are available from the corresponding author upon reasonable request. No other new experimental datasets were generated for this review.

## Supplementary Materials

The following supporting information can be downloaded at https://www.mdpi.com/article/10.3390/biom16060839/s1. Table S1. Detailed information on fish *mc4r* genes. Table S2. Detailed information on fish *mc3r* genes.

## Abbreviations

The following abbreviations are used in this manuscript:

MCR	melanocortin receptor
MC1R	melanocortin-1 receptor
MC2R	melanocortin-2 receptor
MC3R	melanocortin-3 receptor
MC4R	melanocortin-4 receptor
MC5R	melanocortin-5 receptor
MSH	melanocyte-stimulating hormone
ACTH	adrenocorticotropic hormone
POMC	proopiomelanocortin
AgRP	agouti-related peptide
ASIP	agouti-signaling protein or agouti
GPCR	G protein-coupled receptor
LEAP2	liver-expressed antimicrobial peptide 2
CNS	central nervous system
ARC	arcuate nucleus
VWN	ventromedial nucleus
TMD	transmembrane domain
ECL	extracellular loop
ICL	intracellular loop
SNP	single-nucleotide polymorphism
WT	wild type
cAMP	cyclic adenosine monophosphate
ICV	intracerebroventricular injection
DEG	differentially expressed gene
FSH	follicle-stimulating hormone
LH	luteinizing hormone
GnRH	gonadotropin-releasing hormone
PRL	prolactin
MRAP	melanocortin 2-receptor accessory protein
PKA	protein kinase A
PKC	protein kinase C
AC	adenylyl cyclase
PLC	phospholipase C
PI3K	phosphatidylinositol 3-kinase
AKT	protein kinase B
JNK	c-Jun N-terminal kinase
AMPK	AMP-activated protein kinase
PVH	paraventricular nucleus of the hypothalamus
POC	proopiocortin
POM	proopiomelanotropin
